# IL-17 induced NOTCH1 activation in oligodendrocyte progenitor cells enhances proliferation and inflammatory gene expression

**DOI:** 10.1038/ncomms15508

**Published:** 2017-05-31

**Authors:** Chenhui Wang, Cun-Jin Zhang, Bradley N. Martin, Katarzyna Bulek, Zizhen Kang, Junjie Zhao, Guanglin Bian, Julie A. Carman, Ji Gao, Ashok Dongre, Haibo Xue, Stephen D. Miller, Youcun Qian, Dolores Hambardzumyan, Tom Hamilton, Richard M. Ransohoff, Xiaoxia Li

**Affiliations:** 1 Key Laboratory of Molecular Biophysics of the Ministry of Education, College of Life Science and Technology, Huazhong University of Science and Technology, Wuhan 430074, China; 2 Department of Immunology, Lerner Research Institute, Cleveland Clinic Foundation, Cleveland, Ohio 44195, USA; 3 Wuhan Institute of Biotechnology, Wuhan 430075, China; 4 Department of Neurology and Immunology, Tianjin Neurological Institute, Tianjin Medical University General Hospital, Tianjin 300052, China; 5 Department of Pathology, Case Western Reserve University, School of Medicine, Cleveland, Ohio 44106, USA; 6 Shanghai Institute of Immunology, Shanghai Jiaotong University of School of Medicine, 280 South Chongqing Rd, Huangpu, Shanghai 200025, China; 7 Discovery Biology, Bristol-Myers Squibb, Princeton, New Jersey 08540, USA; 8 The Department of Endocrinology and Metabolism, Binzhou Medical University Hospital. Binzhou City, Shandong Province 256603, China; 9 Department of Microbiology-Immunology, Feinberg School of Medicine, Northwestern University, Chicago, Illinois 60611, USA; 10 The Key Laboratory of Stem Cell Biology, Institute of Health Sciences, Shanghai Institutes for Biological Sciences, Chinese Academy of Sciences/Shanghai Jiao Tong University School of Medicine, Shanghai 200031, China; 11 Shanghai Institute of Rheumatology, Shanghai Renji Hospital, Shanghai Jiaotong University School of Medicine, Shanghai 200001, China; 12 Department of Pediatrics, Aflac Cancer and Blood Disorders Center, Emory University. 201 Dowman Drive. Atlanta, Georgia 30322 USA; 13 Biogen Idec, Cambridge, Massachusetts 02142, USA

## Abstract

NOTCH1 signalling contributes to defective remyelination by impairing differentiation of oligodendrocyte progenitor cells (OPCs). Here we report that IL-17 stimulation induces NOTCH1 activation in OPCs, contributing to Th17-mediated demyelinating disease. Mechanistically, IL-17R interacts with NOTCH1 via the extracellular domain, which facilitates the cleavage of NOTHC1 intracellular domain (NICD1). IL-17-induced NOTCH1 activation results in the interaction of IL-17R adaptor Act1 with NICD1, followed by the translocation of the Act1–NICD1 complex into the nucleus. Act1–NICD1 are recruited to the promoters of several NOTCH1 target genes (including STEAP4, a metalloreductase important for inflammation and cell proliferation) that are specifically induced in the spinal cord by Th17 cells. A decoy peptide disrupting the IL-17RA–NOTCH1 interaction inhibits IL-17-induced NOTCH1 activation and attenuates Th17-mediated experimental autoimmune encephalitis (EAE). Taken together, these findings demonstrate critical crosstalk between the IL-17 and NOTCH1 pathway, regulating Th17-induced inflammatory and proliferative genes to promote demyelinating disease.

## Abstract (web summary)

NOTCH signalling stimulates oligodendrocyte progenitor cell proliferation but how this regulates demyelinating disease is unclear. Here, the authors show that an IL-17 adaptor protein, Act1, interacts with the C-terminal fragment of NOTCH1 (NICD) to activate cell proliferation and an inflammatory response.

Multiple sclerosis (MS) is an inflammatory demyelinating disease of the central nervous system (CNS) that exhibits the histopathologic hallmarks of inflammation, demyelination and neurodegeneration^1^. While the precise mechanisms of MS pathogenesis have not been fully elucidated, current models posit that myelin-reactive T helper cell populations play a central role in the initiation and propagation of the pathological process^2^,^3^,^4^. Experimental autoimmune encephalomyelitis (EAE) is a widely used animal model of MS, and elegant studies employing this model have defined the series of pathogenic events that occur in different phases of EAE/MS development^5^,^6^. In the initiation stage of EAE, antigen-presenting cells in the draining lymph nodes are activated and produce cytokines that regulate the differentiation and proliferation of effector CD4 T cells, including the T helper 1 (Th1) and T helper 17 (Th17) cell lineages. Th1 cells are functionally defined by their production of IFN-γ and TNF-α, while Th17 cells produce IL-17, IL-21 and IL-22 (refs 7, 8). It was recently reported that auto-reactive Th1 and Th17 cells are capable of independently inducing EAE through what appears to be distinct effector mechanisms^9^,^10^. Th17 cells are generated as a discrete lineage when naive CD4^+^ T cells are activated in the presence of transforming growth factor β (TGF-β) and IL-6, and they acquire the ability to rapidly expand in the presence of IL-23 (refs 11, 12, 13). While Th17 cells are known to produce a number of key pro-inflammatory cytokines, IL-17 signalling is required for the effector stage of Th17-mediated EAE because genetic ablation of either IL-17 or IL-17 receptor renders mice resistant to EAE development^14^,^15^. However, the precise cellular and molecular basis by which IL-17 participates in the pathogenesis of MS/EAE is still unclear.

Act1 is a key adaptor molecule in the IL-17 signalling pathway, and propagates IL-17 downstream signalling events following ligand stimulation^16^,^17^. We previously reported that deletion of Act1 from the neuroectodermal lineage in mice (neurons, oligodendrocytes and astrocytes) results in attenuated severity of EAE^18^. We examined the cellular basis of this observation. The disease course of EAE was unaffected by deletion of Act1 in neurons or mature oligodendrocytes, and Act1 deletion in astrocytes only modestly affected the disease course. Deletion of Act1 in oligodendrocyte progenitor cells (OPCs) resulted in markedly reduced EAE severity^19^. While IL-17 induced characteristic inflammatory mediator expression in OPCs, IL-17 also exhibited strong inhibitory effects on the maturation of oligodendrocyte lineage cells *in vitro*^19^. These data identify OPCs as the major CNS cellular target of IL-17 in EAE.

The NOTCH signalling pathway is an evolutionarily conserved pathway that regulates developmental processes in both invertebrates and vertebrates^20^,^21^. The majority of studies demonstrating the involvement of the NOTCH pathway in the pathogenesis of EAE have focused on the role of NOTCH in regulating T helper cell maturation and effector cell differentiation^22^,^23^,^24^,^25^. The NOTCH pathway has been shown to control OPC differentiation and proliferation. Specifically, NOTCH activation in OPCs was shown to contribute to defective remyelination in the CNS by impairing the differentiation of OPCs into mature oligodendrocytes^26^. Interestingly, our previous study showed that selective Act1 deficiency in OPCs (NG2^+^/Olig2^+^) confers protection against EAE, and IL-17 treatment *in vitro* inhibits OPC differentiation and reduces OPC survival^19^. However, the precise molecular mechanism mediating this effect has remained undefined. Here we report that IL-17 treatment of OPCs co-cultured with astrocytes leads to NOTCH1 activation in OPCs and that this novel signalling cascade is dependent on the extracellular domains of both IL-17 receptor and NOTCH1. IL-17-induced NOTCH1 activation resulted in formation of the Act1–NICD1 complex, translocation of Act1–NICD1 to the nucleus and recruitment of Act1 and the transcription factor RBP-J to the promoters of several NOTCH target genes that are important for inflammation and cell proliferation. As a result, IL-17-induced NOTCH1 activation in OPCs promoted the inflammatory response, cell proliferation and inhibited OPC maturation. Further underlining the pathogenic significance of IL-17-induced NOTCH1 activation, selective genetic ablation of either NOTCH1 or RBP-J in OPCs was sufficient to greatly attenuate the development and severity of Th17- but not Th1-mediated EAE. Moreover, intracerebroventricular injection of a decoy peptide based on the sequence of IL-17RA greatly inhibited IL-17-induced NOTCH1 activation and attenuated Th17-mediated EAE.

## Results

### Act1 directly interacts with NOTCH1 via NICD1

**Figure 1:**
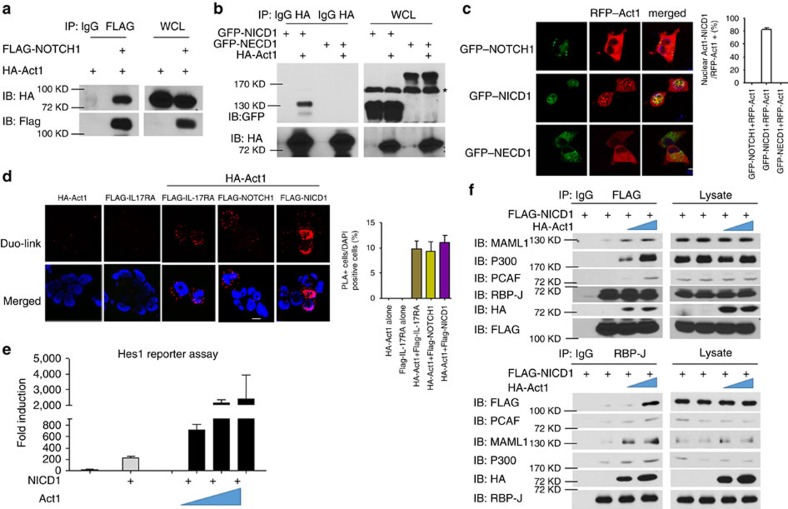
Act1 directly interact with NOTCH1 through NICD1. (**a**) HEK293 cells were transfected with HA–Act1 alone or in combination with FLAG-NOTCH1. Cell lysates were immunoprecipitated with anti-FLAG antibody, followed by immunoblot analysis for indicated proteins. (**b**) HEK293 cells were transfected with GFP–NICD1, GFP–NECD1 and HA–Act1 alone or in combination as indicated. Cell lysates were immunoprecipitated with anti-HA antibody, followed by immunoblot analysis for the indicated proteins. Asterisk indicates non-specific band. (**c**) HeLa cells were transfected with GFP–NOTCH1, GFP–NICD1, GFP–NECD1 and RFP–Act1 as indicated. Images were acquired using confocal microscopy under a × 60 objective; scale bar, 20 μm. Frequencies of cells showing Act1–NICD co-localization in the nuclei in the total RFP-positive cells are shown in bar graphs. (**d**) HeLa cells were transfected with HA–Act1 alone or in combination with indicated plasmids. *In situ* PLA was performed by using rabbit anti-FLAG and mouse anti-HA antibodies followed by proximity ligation (see Methods section) and DAPI staining. Red: PLA signal indicating protein–protein interaction; blue: nuclei. Images were acquired using confocal microscopy under a × 60 objective; scale bar, 20 μm. Frequencies of PLA-positive cells are shown in bar graphs. (**e**) HeLa cells were transfected with Hes1-luciferase reporter (100 ng) alone or with indicated combinations of human NICD cDNA (200 ng) and increasing amounts of human Act1 cDNA (0, 100, 200 and 500 ng). Data are plotted as fold induction of luciferase activity from cells with indicated transfection over that of the Hes1-luciferase transfection alone. (**f**) HEK293 cells were transfected with FLAG–NICD1 (3 μg) with increasing amounts of HA–Act1 (3 and 6 μg). Cell lysates were immunoprecipitated with anti-FLAG (upper panel) or anti-RBP-J antibody (lower panel), followed by immunoblot analysis for the indicated antibodies. IgG, immunoglobulin G; IB, immunoblotting; IP, immunoprecipitation; WCL, whole-cell lysates. Arrow indicates the band for FLAG–NICD1. *P<0.05 based on Mann–Whitney *U*-test. All error bars represent s.e.m. of technical replicates. Data are representative of three independent experiments.

To identify novel interacting partners of Act1, we immunoprecipitated endogenous Act1 in the lysates of Hela cells treated with IL-17, followed by mass spectrometric analysis of the proteins that were co-immunoprecipitated with Act1. Several NOTCH family member proteins were detected, including NOTCH1, NOTCH2 and NOTCH3 (Supplementary Fig. 1). Since Act1 interacted most strongly with NOTCH1, we decided to focus on the Act1–NOTCH1 interaction. The interaction of Act1 with NOTCH1 was confirmed by co-immunoprecipitation followed by western blot analysis (Fig. 1a). Structure-function analysis indicated that Act1 interacts with the intracellular domain (NICD1) but not the extracellular domain (NECD1) of NOTCH1 (Fig. 1b). We further examined the Act1–NICD1 interaction by imaging analysis following transfection with GFP- or RFP-tagged proteins (Fig. 1c). When co-expressed with NECD1 (GFP), Act1 (RFP) was primarily located in the cytoplasm and did not co-localize with NECD1. In dramatic contrast, Act1 co-localized with NICD1 in the nucleus (Fig. 1c). To visualize the interaction between Act1 and NOTCH1/NICD1, we performed an *in situ* proximity ligation assay (PLA) using IL-17RA as a positive control. We detected a strong signal for the Act1–NOTCH1 complex in the cytoplasm and the Act1–NICD1 interaction in the nucleus (Fig. 1d).

On ligand binding, the NOTCH1 receptor is cleaved by ADAM family metalloproteases followed by the intramembrane γ-secretase complex to generate NICD1, which translocates into the nucleus to convert the DNA-binding protein RBP-J from a transcriptional repressor into an activator. This process involves the formation of a stable complex consisting of NICD1, RBP-J and Mastermind-like family of co-activators (MAML), and it serves as a platform for the further recruitment of other co-activators to activate NOTCH1 target genes^20^,^21^. Given our finding that Act1 could bind directly to NICD1, we further tested whether Act1 had any impact on the NOTCH1 pathway. We found that Act1 was able to increase NOTCH1 target gene Hes1-driven reporter activity in a dose-dependent manner when co-expressed with NICD1 (Fig. 1e), suggesting that Act1 might play a positive role in the NOTCH1 pathway through its interaction with NICD1. Since Act1 is a U-box-containing E3 ligase^27^ and NICD1 is known to be regulated by ubiquitination^28^,^29^, we next examined whether the E3 ligase activity of Act1 is required for its impact on NICD1 activity. We found that the Act1 E3 mutant (ΔU-box) lost the ability to translocate into the nucleus (Supplementary Fig. 2a) due to defective binding to NICD1 (Supplementary Fig. 2b). As a result, the Act1 E3 mutant failed to promote NICD1 activity compared with wild-type Act1 (Supplementary Fig. 2c). Interestingly, Act1 induced K63-linked polyubiquitination of NICD1 (Supplementary Fig. 2d). Taken together, these results suggest that the E3 ligase activity is required for its ability to promote NICD1 activity, probably by ubiquitinating NICD1. Since the Act1 E3 mutant (ΔU-box) failed to interact with NICD1, Act1-mediated NICD1 ubiquitination might be critical for the stability of the Act1–NICD1 complex. To further understand the mechanism by which Act1 facilities NICD1 activity, we examined the possible impact of Act1 on NICD1–RBP-J complex formation and the recruitment of co-activators including MAML1, P300 and PCAF. Interestingly, Act1 was detected in the RBP-J–NICD1 complex, and overexpression of Act1 increased the recruitment of MAML1, P300 and PCAF to the Act1–NICD1–RBP-J complex (Fig. 1f). Collectively, these findings suggest that Act1 directly binds to NOTCH1 through NICD1 and ubiquitinates NICD1 to form a stable complex with NICD1–RBP-J in the nucleus, facilitating the recruitment of co-activators for gene transcription.

### IL-17 activates the NOTCH pathway in OPC-astrocyte co-cultures

**Figure 2:**
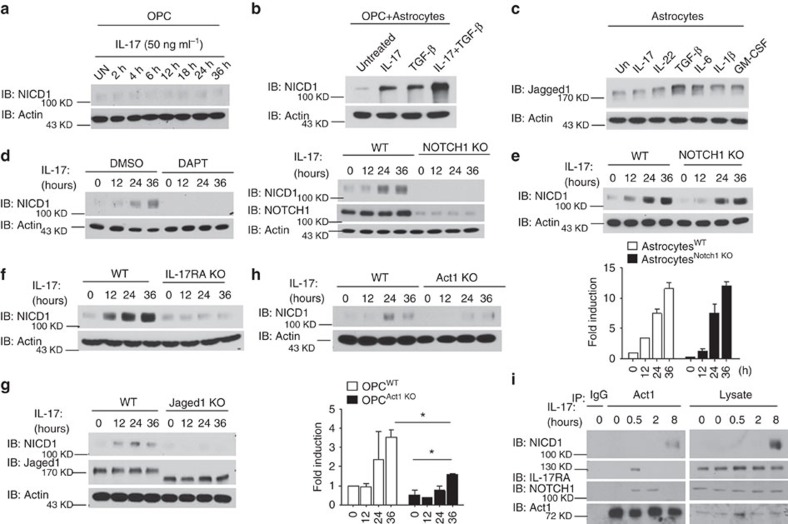
IL-17 activates NOTCH pathway in OPCs co-cultured with astrocytes. (**a**) OPCs were stimulated with IL-17 (50 ng ml^−1^) for indicated time, followed by immunoblot analysis for indicated proteins. (**b**) OPC-astrocyte co-cultures were treated with IL-17 (50 ng ml^−1^), TGF-β (10 ng ml^−1^) or IL-17+TGF-β for 24 h, followed by immunoblot analysis for indicated proteins. (**c**) Astrocytes were left untreated or treated with IL-17 (50 ng ml^−1^), IL-22 (10 ng ml^−1^), TGF-β (10 ng ml^−1^), IL-6 (10 ng ml^−1^), IL−1β (1 ug ml^−1^) and GM-CSF (10 ng ml^−1^) for 24 h, followed by immunoblot analysis for Jagged1 and Actin. (**d**) OPC-astrocyte co-cultures were pretreated with dimethylsulfoxide (DMSO) or DAPT (10 μM) for 6 h. Pretreated cells were stimulated with IL-17 (50 ng ml^−1^) for the indicated times, followed by immunoblot analysis (left panel). Co-cultured wild-type or NOTCH1 knockout (KO) OPCs were stimulated with IL-17 (50 ng ml^−1^) for the indicated time, followed by immunoblot analysis (right panel). (**e**) OPCs co-cultured with wild-type or NOTCH1 KO astrocytes were stimulated with IL-17 (50 ng ml^−1^) for the indicated time, followed by immunoblot analysis. Densitometric quantification of western blots from two independent experiments is shown as fold induction of NICD1 in IL-17-treated cells over untreated cells. (**f**) Co-cultured wild-type or IL-17RA KO OPCs were stimulated with IL-17 (50 ng ml^−1^) for the indicated times, followed by immunoblot analysis for indicated proteins. (**g**) OPCs co-cultured with wild-type or Jagged1 KO astrocytes were stimulated with IL-17 (50 ng ml^−1^) for the indicated time, followed by immunoblotting analysis. Densitometric quantification is performed as described for **e**. (**h**) Co-cultured wild-type or Act1 KO OPCs were stimulated with IL-17 (50 ng ml^−1^) for the indicated times, followed by immunoblot analysis for indicated proteins. Densitometric quantification of western blots from two independent experiments is shown as fold induction of NICD1 in IL-17-treated cells over untreated cells. (**i**) OPCs co-cultured with Act1 KO astrocytes were stimulated with IL-17 (50 ng ml^−1^) for indicated time. Cell lysates were immunoprecipitated with anti-Act1 antibody, followed by immunoblot analysis. All error bars represent s.e.m. of technical replicates *P<0.05 based on Mann–Whitney *U*-test. Data are representative of three independent experiments.

Our previous study revealed that the pathogenic impact of IL-17 signalling during neuroinflammation is most critical in NG2^+^ OPCs, as selective genetic ablation of Act1 in OPCs but not in other neuroglial populations, ameliorated the Th17-mediated EAE phenotype^19^. In that study, IL-17 was also shown to inhibit OPC differentiation while promoting OPC proliferation through an unclear mechanism^19^. It is well established that the NOTCH1 pathway controls the differentiation and proliferation of OPCs, and NOTCH1 signalling has also been shown to modulate demyelination/remyelination in mouse models of inflammatory demyelinating disease^26^,^30^. Collectively, these findings prompted us to examine the possibility that IL-17 might affect the NOTCH1 pathway in OPCs. Treatment of OPCs with IL-17 for different time periods had no observable impact on NOTCH1 activation, as assessed by production of the NOTCH1 cleavage product NICD1 (Fig. 2a). An earlier study suggested that TGF-β acts on astrocytes to promote expression of the NOTCH1 ligand Jagged1, thereby activating NOTCH1 in OPCs and inhibiting their differentiation^26^. Consistent with the previous report, we detected an increase in NICD1 cleavage in OPCs co-cultured with astrocytes following stimulation with TGF-β. Intriguingly, IL-17 also induced NICD1 cleavage in this co-culture system, and treatment with IL-17+TGF-β resulted in enhanced NICD1 production compared with IL-17 or TGF-β treatment alone (Fig. 2b). One possible explanation for NOTCH1 pathway activation in this system was that IL-17 might upregulate Jagged1 expression in astrocytes, thus indirectly activating NOTCH1 in nearby OPCs. However, analysis of an astrocyte monoculture revealed that while stimulation with TGF-β treatment or IL-6 upregulated expression of Jagged1, there was no change in Jagged1 expression in response to IL-17 treatment (Fig. 2c). We also examined whether IL-17 might upregulate other NOTCH pathway ligands in astrocytes and found that IL-17 did not have any impact on the expression of the other NOTCH ligands (Supplementary Fig. 3). Taken together, these results suggested that IL-17 stimulation might have a direct impact on the activation of NOTCH1 in OPCs co-cultured with astrocytes.

Cleavage of NICD1 in response to NOTCH receptor ligation is a γ-secretase-dependent process. Pre-treatment with DAPT (a γ-secretase inhibitor) abolished IL-17-induced cleavage of NICD1 at all tested time points, suggesting that IL-17-induced NOTCH activation is also γ-secretase dependent (Fig. 2d, left panel). Notably, IL-17-induced NICD1 cleavage was abolished in a co-culture containing NOTCH1 KO OPCs, suggesting that IL-17-induced NOTCH1 activation is intrinsic to OPCs (Fig. 2d, right panel). Supporting this finding, co-culture of wild-type OPCs with NOTCH1 KO astrocytes had no significant impact on IL-17-induced NICD cleavage (Fig. 2e). Likewise, NOTCH1 activation was completely abolished in a co-culture containing IL-17RA KO OPCs (Fig. 2f). However, consistent with the observation that IL-17-induced NOTCH1 activation in OPCs required astrocyte co-culture, IL-17-induced NICD cleavage was completely abolished in the co-culture of OPCs with Jagged1 KO astrocytes, indicating that astrocytes expressing Jagged1 were indispensable for IL-17-induced NOTCH activation in OPCs (Fig. 2g).

Given that Act1 directly interacts with the intracellular domain of NOTCH1, the next question was whether Act1 is required for IL-17-induced NICD1 cleavage. Surprisingly, Act1 deficiency in OPCs co-cultured with astrocytes resulted in a partial defect in NICD1 cleavage in response to IL-17 treatment, which suggested that IL-17-induced NICD1 cleavage was mediated mostly at the receptor level and that Act1 played only a partial role in this process (Fig. 2h). Interestingly, we found that while Act1 was recruited to IL-17RA and NOTCH1 shortly after IL-17 stimulation (30 min), the NICD1–Act1 complex was only detected at 8 h after IL-17 stimulation (Fig. 2i). The γ-secretase-dependent process probably accounted for the delay in NOTCH1 cleavage after the IL-17-induced interaction of IL-17R–Act1 with NOTCH1. The interaction of Act1 with IL-17RA was more transient than its interaction with NOTCH1, implicating that Act1 may stay with NOTCH1 to eventually form the Act1–NICD1 complex.

### IL-17RA–NOTCH1 interaction induces NICD cleavage

**Figure 3:**
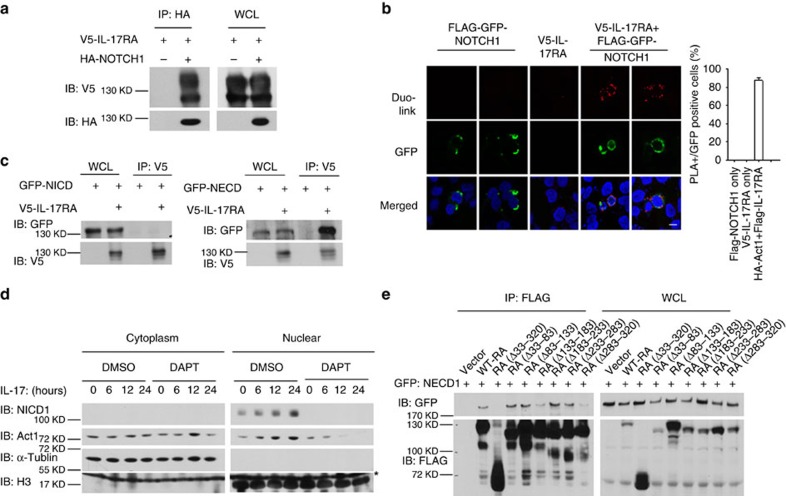
IL-17 activate NOTCH pathway through direct interaction between NOTCH1 and IL-17R. (**a**) HEK293 cells were transfected with V5-IL-17RA alone or in combination with HA–NOTCH1. Cell lysates were immunoprecipitated with anti-HA antibody, followed by immunoblot analysis for indicated proteins. (**b**) HeLa cells were transfected with FLAG–GFP–NOTCH1 and V5-IL-17RA alone or in combination as indicated. *In situ* PLA were performed using rabbit anti-FLAG and mouse anti-V5 antibodies, followed by *in situ* proximity ligation and DAPI staining. Green: GFP (NOTCH1); red: PLA signal; blue: nuclei. Images were acquired using confocal microscopy under a × 60 objective; scale bar, 20 μm. Frequencies of PLA-positive cells in GFP-positive cells are shown in bar graph. (**c**) HEK293 cells were transfected with GFP–NICD1 (left panel) or NECD1 (right panel) with or without V5-IL-17RA. Cell lysates were immunoprecipitated with anti-V5 antibody, followed by immunoblot analysis for indicated proteins. (**d**) OPCs co-cultured with IL-17RA KO astrocytes were pretreated with DMSO or DAPT (10 μM) for 6 h. Pretreated cells were then stimulated with IL-17 (50 ng ml^−1^) for indicated times, followed by cytoplasm-nucleus fractionation. Cell fractionations were analysed by immunoblot for indicated proteins. Arrow indicates H3 bands and asterisk indicates non-specific band. (**e**) HEK293 cells were transfected with GFP–NECD1 in combination with vector or FLAG-tagged IL-17RA deletion mutants as indicated. Cell lysates were immunoprecipitated with anti-FLAG antibody, followed by immunoblot analysis for indicated proteins. IB, immunoblotting; IP, immunoprecipitation; WCL, whole-cell lysates. Error bars represent s.e.m. of technical replicates *P<0.05 based on Mann–Whitney *U*-test. Data are representative of three independent experiments.

We found that IL-17-induced NOTCH activation required NOTCH1, IL-17 receptor and the NOTCH ligand Jagged1 (Fig. 2d,f,g), suggesting that this activation might be initiated at the receptor level. We next investigated whether there was any interaction between IL-17 receptor and NOTCH1. Co-immunoprecipitation and *in situ* PLA revealed that IL-17RA indeed formed a complex with NOTCH1 (Fig. 3a,b), and this interaction required the extracellular domain of NOTCH1 (NECD1) (Fig. 3c). Thus, we hypothesized that the interaction of IL-17R and NOTCH1 via the extracellular domain facilitates the cleavage of NICD1; Act1–NICD1 then forms a complex and translocates into the nucleus. In support of this hypothesis, IL-17 stimulation indeed induced nuclear translocation of NICD1 and Act1 in OPC-astrocyte co-culture (wild-type OPCs with IL-17RA KO astrocytes), which was blocked by N-[(3,5-Difluorophenyl)acetyl]-L-alanyl-2-phenyl]glycine-1,1-dimethylethyl ester (DAPT) (a γ-secretase inhibitor) (Fig. 3d). This result suggested that the cleavage of NICD1 was necessary for Act1–NICD1 nuclear translocation. Consistently, while Jagged1 expressed by astrocytes was required for IL-17-induced NOTCH1 cleavage in the co-culture system, NICD1 and Act1 nuclear translocation were abolished in OPCs co-cultured with Jagged1 KO astrocytes (Supplementary Fig. 4).

The finding that IL-17RA directly interacted with NOTCH1 prompted us to carefully analyse the protein regions mediating this interaction. We found that deletion of the extracellular domain of IL-17RA (IL-17RA Δ33–320) completely abolished the interaction between IL-17RA and NECD1, indicating that the IL-17R–NOTCH1 interaction occurred via their extracellular domains (Fig. 3e). Furthermore, sequential deletion analysis of IL-17RA demonstrated that removal of two small regions within the extracellular domain of IL-17RA (Δ133–183; Δ283–320) greatly reduced the IL-17RA–NECD1 interaction (Fig. 3e).

### NICD1–Act1 translocates into the nucleus of OPCs during EAE

**Figure 4:**
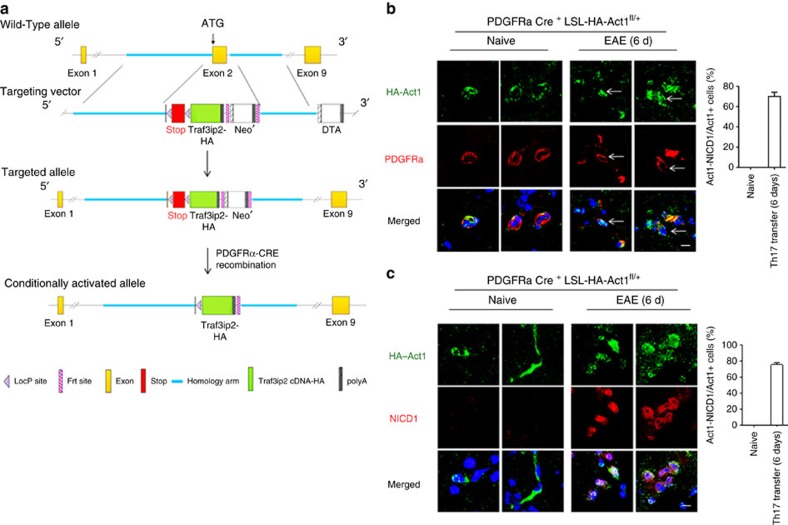
Th17 adoptive transfer induces NOTCH1 pathway activation and NICD1–Act1 translocation to the nucleus of OPCs *in vivo*. (**a**) Design and generation of the LSL–HA–Act1 knock-in mice. See the experimental procedure for the details. (**b**,**c**) *PDGFRα-CRE*^*+*^ *LSL–HA–Act1* mice were left untreated (naive) or transferred with MOG-reactive Th17 cells to induce EAE. Mice were killed 6 days later. Frozen sections of brain tissue from experimental mice were stained to visualize HA–Act1, PDGFRα (**b**) or NICD (**c**). Images were acquired using confocal microscopy under a × 60 objective. Scale bar, 10 μm. Frequencies of cells showing Act1–NICD co-localization in Act1-positive cells are shown in bar graph. Error bar represents s.e.m. of biological replicates (mice). *P<0.05 based on Mann–Whitney *U*-test. Data are representative of three independent experiments.

To examine whether IL-17 could induce NOTCH1 activation and Act1–NICD1 nuclear translocation *in vivo*, we generated a floxed HA-tagged Act1 knock-in mouse (into its own locus), which enabled us to track endogenous Act1 cellular localization *in vivo* in a cell-type-specific manner (Fig. 4a). By breeding this mouse line to a PDGFRα-CRE (specifically expressed in OPCs) transgenic mouse, we obtained an OPC-specific HA–Act1 knock-in mouse line (Fig. 4b), which was used to track NOTCH1 activation and Act1 cellular localization in the CNS system in a model of Th17 adoptive transfer-induced EAE (Fig. 4a). By immunofluorescence staining, we observed that Act1 was mainly expressed in the cytoplasm of OPCs (PDGFRα^+^ cells) in the brain of naive mice (Fig. 4b). Interestingly, 6 days after Th17 adoptive transfer, Act1 was predominantly localized in the nuclei of OPCs (PDGFRα^+^ cells) (Fig. 4b). Moreover, we detected robust NICD1 staining in the nuclei of OPCs and perfect Act1–NICD1 nuclear co-localization (Fig. 4c). These data provided *in vivo* evidence for the function of the IL-17/Act1/NOTCH1 axis in OPCs during the course of EAE.

### IL-17 induces a subset of NOTCH1-dependent genes in OPCs

**Figure 5:**
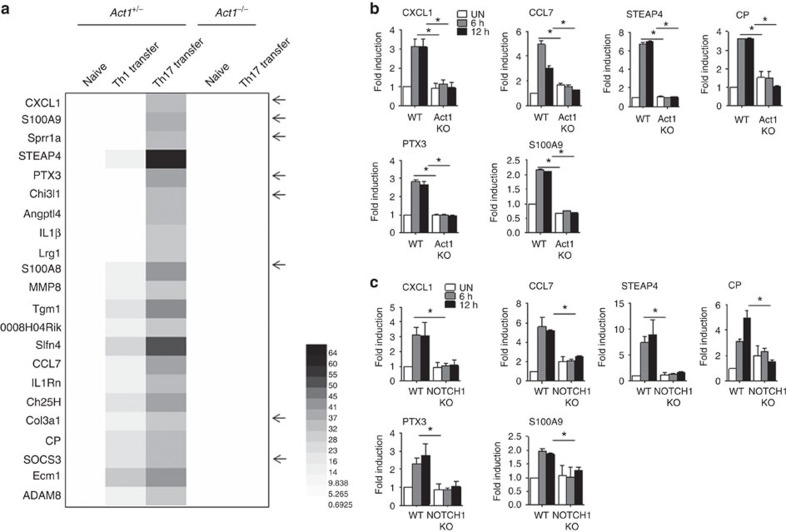
IL-17 induces NOTCH1-dependent genes in co-cultured OPCs. (**a**) Wild-type and Act1 KO mice were left untreated or transferred with Th1 or Th17 cell to induce EAE. At the peak of the disease, mice were killed, and spinal cords were subjected to microarray analysis. Genes specifically induced by Th17 comparing to the spinal cords from naive mice are shown in the heat map (the arrows indicate NOTCH target genes reported in the literature^41^,^42^,^43^). (**b**) Wild-type or Act1 KO OPCs co-cultured with Act1 KO astrocytes were stimulated with IL-17 (50 ng ml^−1^) for indicated time, followed by RT–PCR analysis for indicated genes. (**c**) Wild-type or NOTCH1 KO OPCs co-cultured with Act1 KO astrocytes were stimulated with IL-17 (50 ng ml^−1^) for indicated time, followed by RT–PCR analysis for indicated genes. *P<0.05 based on Mann–Whitney *U*-test. All error bars represent s.e.m. of technical replicates. Data are representative of three independent experiments.

IL-17 activates the NF-κB and MAPK pathways, thereby inducing inflammatory gene expression. Furthermore, IL-17-mediated pro-inflammatory gene expression has been shown to be important in many disease states, including MS/EAE. Given the surprising finding that IL-17 can also induce NOTCH1 activation in OPCs, we sought to determine whether this novel IL-17–NOTCH1 axis plays any role in EAE pathogenesis. Th1- or Th17-polarized myelin oligodendrocyte glycoprotein (MOG)-reactive T cells were adoptively transferred to a naive recipient. At the peak of EAE disease, microarray analysis of gene expression was conducted on recipient spinal cords. Bioinformatics analysis revealed that a number of genes were specifically induced in Th17 cell-recipient spinal cords (Fig. 5a). We found that IL-17 stimulation was able to induce these Th17-specific genes in wild-type OPCs co-cultured with Act1 KO astrocytes, but not in the co-culture of Act1 KO OPCs (Fig. 5b). Interestingly, the induction of these Th17-specific genes was abolished in NOTCH1 KO OPC co-cultures, including the inflammatory genes *CXCL1*, *STEAP4*, *PTX3*, *S100A9*, *CCL7* and *CP* (Fig. 5c). With the exception of STEAP4, all of these gene products have previously been implicated in the pathogenesis of MS/EAE. It is important to note that, compared with the astrocytes-OPC co-culture, IL-17 stimulation of OPCs alone induced much lower levels of expression of the IL-17-induced genes indicated above (Supplementary Fig. 5a). As a control, we showed that TGF-β failed to upregulate these IL-17-induced genes in an astrocyte-OPC co-culture. However, whereas Hes5 and Hes7 were highly induced by TGF-β in a NOTCH1-dependent manner, these two genes were not induced by IL-17 in astrocyte-OPC co-culture (Supplementary Fig. 5b,c).

**Figure 6:**
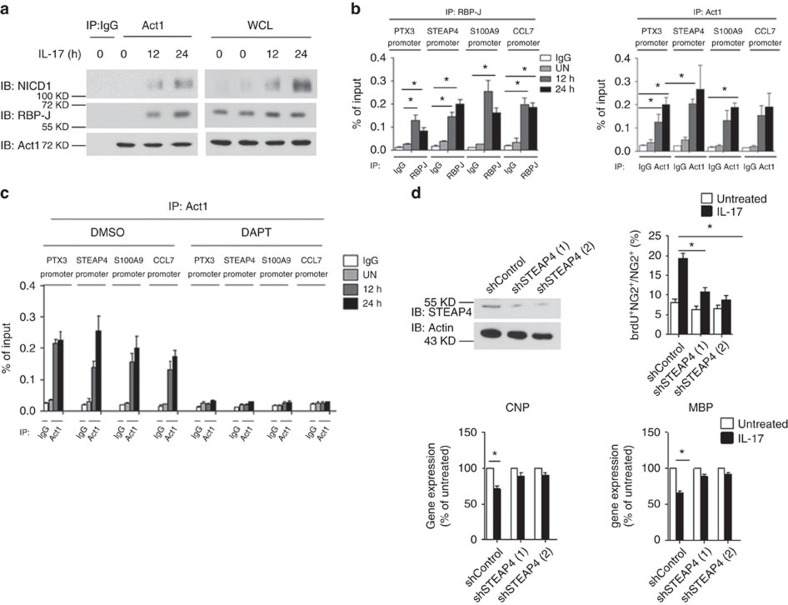
Act1 binds to the promoters of NOTCH1-depdnent genes in response to IL-17 stimulation. (**a**) Wild-type OPCs co-cultured with Act1 KO astrocytes were stimulated with IL-17 (50 ng ml^−1^) for the indicated time. Cell lysates were immunoprecipitated with anti-Act1 antibody, followed by immunoblot. (**b**) Wild-type OPCs co-cultured with Act1 KO astrocytes were stimulated with IL-17 (50 ng ml^−1^) for indicated time. Stimulated cells were subjected to ChIP assay using anti-RBP-J or anti-Act1 antibodies for the enrichment of indicated promoters. (**c**) Wild-type OPCs co-cultured with Act1 KO astrocytes were pretreated with DMSO or DAPT (10 μM) for 6 h. Pretreated cells were stimulated with IL-17 (50 ng ml^−1^) for the indicated time, followed by ChIP assay using RBP-J and Act1 antibodies for indicated promoters. (**d**) NG2^+^ OPCs cells were lentivirally transduced with control shRNA (shControl) or two different shRNAs targeting STEAP4. Efficiency of knockdown was determined by immunoblot analysis. Infected NG2^+^ OPCs cells co-cultured with Act1 KO astrocytes were subjected to BrdU incorporation assay after IL-17 (50 ng ml^−1^) treatment for 24 h. A total of 1,000 NG2^+^ cells were enumerated from 10 different views for BrdU positivity (upper panel). NG2^+^ OPCs cells transduced with control shRNA or shRNA targeting STEAP4 were co-cultured with Act1 KO astrocytes and treated with IL-17 (50 ng ml^−1^) for 24 h. Expressions of CNP and MBP were analysed by RT–PCR (lower panel). IB, immunoblotting; IP, immunoprecipitation; WCL, whole-cell lysates CNP, 2',3'-Cyclic nucleotide 3'-phosphodiesterase; MBP, myelin basic protein. *P<0.05 based on Mann–Whitney *U*-test. All error bars represent s.e.m. of technical replicates. Data are representative of three independent experiments.

Since IL-17 induces the nuclear translocation of the Act1–NICD1 complex, we next questioned whether Act1–NICD1 could form a complex with RBP-J on the promoters of those Th17-induced NOTCH targets. Co-immunoprecipitation showed that IL-17 stimulation indeed induced the interaction of Act1 with NICD1 and RBP-J in OPC co-cultures (wild-type OPCs with Act1 KO astrocytes) (Fig. 6a). Chromatin immunoprecipitation analysis of IL-17-treated OPC co-cultures (wild-type OPCs with Act1 KO astrocytes) demonstrated that RBP-J was recruited to the promoter region of STEAP4, PTX3, S100A9 and CCL7 in response to IL-17 stimulation. However, we did not observe recruitment of RBP-J to the promoter of CXCL1 or CP. These findings suggest that STEAP4, PTX3, S100A9 and CCL7 are direct target genes of the IL-17–NOTCH axis, while CXCL1 and CP expression might be regulated by an indirect mechanism (Fig. 6b). These findings prompted us to examine the recruitment of Act1 to the STEAP4, PTX3, S100A9 and CCL7 promoter regions in IL-17-treated OPCs co-cultures (wild-type OPCs with Act1 KO astrocytes). Indeed, mirroring what we observed with the NOTCH transcription factor RBP-J, Act1 was also recruited to the promoter region of these genes in response to IL-17 treatment (Fig. 6b). Notably, the IL-17-induced recruitment of Act1 to these promoters was completely blocked by the γ-secretase inhibitor DAPT (Fig. 6c), indicating that Act1 binding to the Th17-induced NOTCH target genes was dependent on NOTCH activation/cleavage followed by the NICD1–Act1 interaction and nuclear translocation.

It is important to note that some of the Th17-induced NOTCH target genes, such as *STEAP4* and *S100A9*, actually have dual roles in inflammatory and proliferative responses^31^,^32^,^33^,^34^,^35^. STEAP4, a metalloreductase with NADPH oxidase (NOX) activity, has been reported to play a role in cell metabolism and cell proliferation. IL-17-induced STEAP4 expression was indeed found to promote keratinocyte proliferation^31^. Thus, we wondered whether STEAP4 might also play a role in OPC proliferation and differentiation. We knocked down STEAP4 expression in OPCs by lentiviral shRNA, and then examined cell proliferation and differentiation in co-culture with IL-17RA KO astrocytes. STEAP4 knockdown attenuated IL-17-induced OPC proliferation and reversed IL-17-mediated inhibition of OPC differentiation (Fig. 6d). These data suggest that the role of IL-17 in OPC proliferation and differentiation might be at least partially dependent on STEAP4 induction.

### Ablation of NOTCH1 in OPCs ameliorates Th17-induced EAE

**Figure 7:**
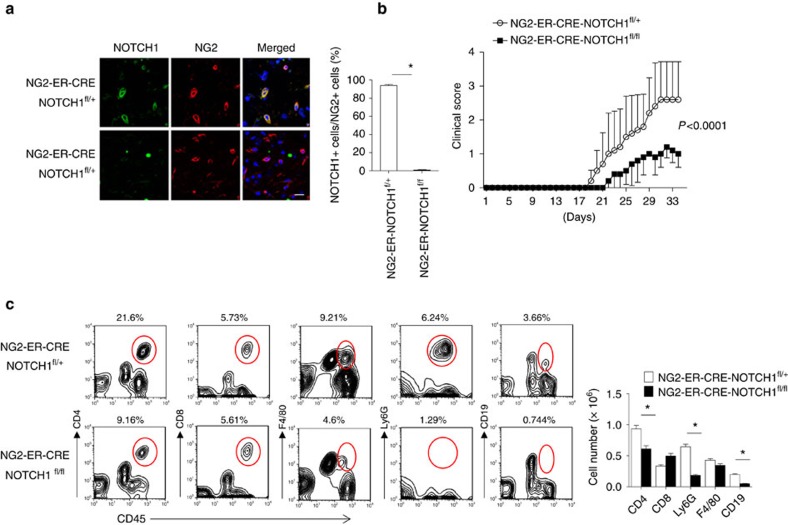
NOTCH1 deficiency in NG2^+>^ OPCs attenuates Th17-induced EAE. (**a**) Transversal sections of lumbar spinal cords from mice of indicated genotypes (*n*=5) were stained with anti-NG2 (red) and anti-NOTCH1 (green) antibodies. Images were then acquired using confocal microscopy under a × 60 objective; scale bar, 20 μm. Frequencies of NOTCH1 NG2^+^ cells were determined to assess deletion efficiency. (**b**) Mice of indicated genotypes were adoptively transferred with MOG-reactive Th17 cells (*n*=5) to induce EAE. Spinal cords were collected at peak of disease. Clinical scores of EAE symptoms in mice described for **a** are graphed over the experimental time course. (*P*<0.0001 based on two-way ANOVA). (**c**) Infiltrating cells in the brains of mice with Th17-induced EAE (*n*=5) were isolated at the peak of the disease, followed by flow cytometry analysis. The numbers of different infiltrating cells were calculated for each mouse. All error bars represent s.e.m. of biological replicates. *P<0.05 based on Mann–Whitney *U*-test. Data are representative of three independent experiments.

**Figure 8:**
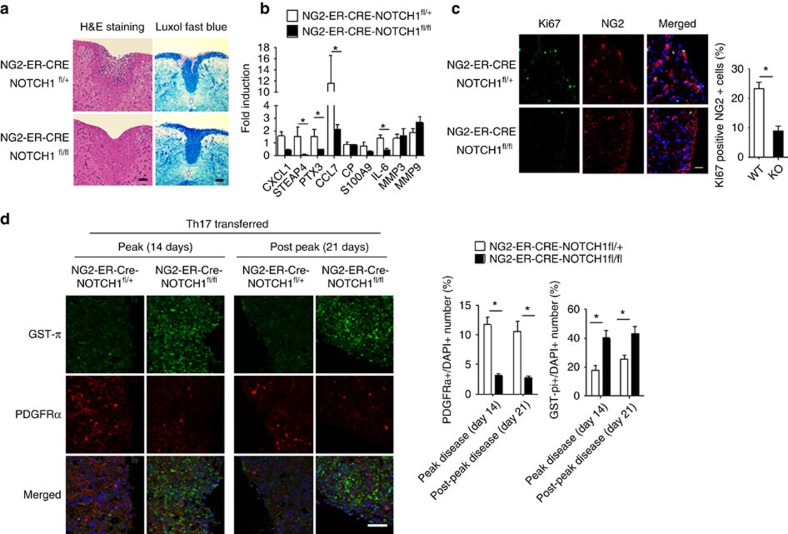
NOTCH1 deficiency in NG2^+^ OPCs reduces cell proliferation and inflammatory gene expression. (**a**) Haematoxylin and LFB staining of transversal sections of lumbar spinal cords from mice with Th17-induced EAE. Scale bars, 50 μm (left panel), 100 μm (right panel). (**b**) RT–PCR analysis of inflammatory gene expression in spinal cords from EAE mice (*n*=5) of indicated genotypes. (**c**) Transversal frozen sections of lumbar spinal cords described for **b** were stained with anti-NG2 (red) and anti-Ki67 (green) antibodies. The number of total NG2^+^ cells and Ki67^+^ NG2^+^ double-positive cells were enumerated from three inconsecutive sections from the same spinal cord. Average percentage of Ki67^+^ NG2^+^ cells were calculated. Means of the percentage of each genotype (*n*=5) are plotted. Images were acquired using confocal microscopy with a × 60 objective; scale bar, 40 μm. (**d**) Spinal cords from indicated mice were collected 14 days or 21 days after the peak of Th17-induced EAE. Transversal sections of lumbar spinal cords were stained with anti-GST-π (green) and anti-PDGFRα (red) antibodies. Frequency of GST-π-positive and PDGFRα-positive cells were determined by manual determination. Error bars represent s.e.m. of biological replicates. *P<0.05 based on Mann–Whitney *U*-test. Data are representative of two independent experiments.

The IL-17–NOTCH axis-induced genes identified in this study have previously been implicated in the pathogenesis of MS/EAE^36^,^37^. Therefore, we hypothesized that OPC-intrinsic NOTCH1 might critically impact Th17-mediated EAE development. To test this hypothesis, we generated *NG2*^*ER-Cre*^*NOTCH1*^*f/+*^ and *NG2*^*ER-Cre*^*NOTCH1*^*f/f*^ mice to specifically ablate NOTCH1 expression in NG2^+^ OPCs. Immunofluorescence confirmed the efficient deletion of NOTCH1 in NG2^+^ OPC cells of the spinal cord after induction of NOTCH1 depletion by tamoxifen. Interestingly, it seemed that all the NG2^+^ OPC cells expressed NOTCH1 based on immunofluorescent staining (Fig. 7a). MOG-reactive Th17 cells were then adoptively transferred into sublethally irradiated *NG2*^*ER-Cre*^*NOTCH1*^*f/+*^ and *NG2*^*ER-Cre*^*NOTCH1*^*f/f*^ recipient mice, which showed that the severity of Th17 cell-induced EAE was greatly reduced in *NG2*^*ER-Cre*^*NOTCH1*^*f/f*^ mice compared with the control mice (Fig. 7b). Analysis of brain-infiltrating mono nucleus cell populations revealed that *NG2*^*ER-Cre*^*NOTCH1*^*f/f*^ mice had fewer infiltrating CD4^+^ cells, macrophages, neutrophils and B cells compared with the control mice (Fig. 7c). Histopathological analysis mirrored these findings, with reduced inflammatory infiltration and demyelination observed in spinal cords from *NG2*^*ER-Cre*^*NOTCH1*^*f/f*^ mice (Fig. 8a). Notably, the expression of genes that were directly controlled by the IL-17–NOTCH1 axis (CXCL1, STEAP4 and PTX3) was greatly reduced in spinal cords from *NG2*^*ER-Cre*^*NOTCH1*^*f/f*^ recipients compared with the controls (Fig. 8b).

In a previous study, we reported that following adoptive transfer of MOG-reactive Th17 cells, the NG2^+^ OPC cell population was expanded in the CNS of wild-type mice, but not in *NG2*^*Cre*^*Act1*^*f/*^ mice^19^. Therefore, we speculated that IL-17-induced NOTCH1 activity might have contributed to this phenotype since the NOTCH pathway is known to promote OPC proliferation and interfere with OPC differentiation^26^. During Th17-mediated EAE, the percentage of Ki67^+^NG2^+^ and PDGFRα^+^ OPC cells was significantly higher in the spinal cords of control mice than in *NG2*^*ER-Cre*^*NOTCH1*^*f/f*^ mice (Fig. 8c,d). These results suggest that IL-17–Act1–NOTCH1 signalling may promote OPC proliferation, thereby attenuating the proper maturation of OPCs required for the remyelination process after Th17-induced demyelination. In support of this finding, the matured oligodendrocytes (GST-π^+^ cells) were indeed highly reduced in the control mice compared to with the *NG2*^*ER-Cre*^*NOTCH1*^*f/f*^ mice (Fig. 8d). To test whether NOTCH activation plays a specific role in Th17-mediated EAE, we adoptively transferred MOG-reactive Th1 cells into sublethally irradiated *NG2*^*Cre*^*NOTCH1*^*f/+*^ *and NG2*^*ER-Cre*^*NOTCH1*^*f/f*^ recipient mice. NG2-specific NOTCH1 deletion did not result in a noticeable impact on TH1-induced EAE, including the clinical score, cell infiltration or gene expression, further highlighting the role of NOTCH activation in the IL-17 pathway in OPCs (Supplementary Fig. 6).

In addition to NOTCH1, we also found that Act1 co-immunoprecipitated with NOTCH2 and NOTCH3 based on MS spectrum identification (Supplementary Fig. 1). Additionally, microarray analysis revealed that NOTCH2 was also induced in the spinal cords of mice receiving MOG-reactive Th17 cells (Supplementary Data 1). Given that RBP-J is a common transcription factor for all NOTCH members, we generated *NG2*^*ER-Cre*^*RBP-J*^*f/+*^ and *NG2*^*ER-Cre*^*RBP-J*^*f/f*^ mice. Western blot analysis showed that the expression of RBP-J was completely ablated in NG2^+^ OPC cells derived from *NG2*^*Cre*^*RBP-J*^*f/f*^ (Supplementary Fig. 7a). Following the adoptive transfer of MOG-reactive Th17 cells, the EAE phenotype was dramatically reduced in *NG2*^*ER-Cre*^*RBP-J*^*f/f*^ mice compared with the control mice (Supplementary Fig. 7b). The impact of NG2-specific *RBP-J* deletion on Th17-induced EAE was more substantial than that of NG2-specific *NOTCH1* deletion, implicating possible involvement of NOTCH2/3 in this IL-17–NOTCH axis during Th17-induced EAE. However, NG2-specific *RPB-J* deletion did not have a noticeable impact on Th1-induced EAE (Supplementary Fig. 7c). Together, these data indicated that NOTCH pathways other than NOTCH1 might also play a crucial role in the pathogenesis of Th17-, but not Th1-mediated, EAE.

### Disruption of the IL-17RA–NOTCH1 interaction attenuates EAE

**Figure 9:**
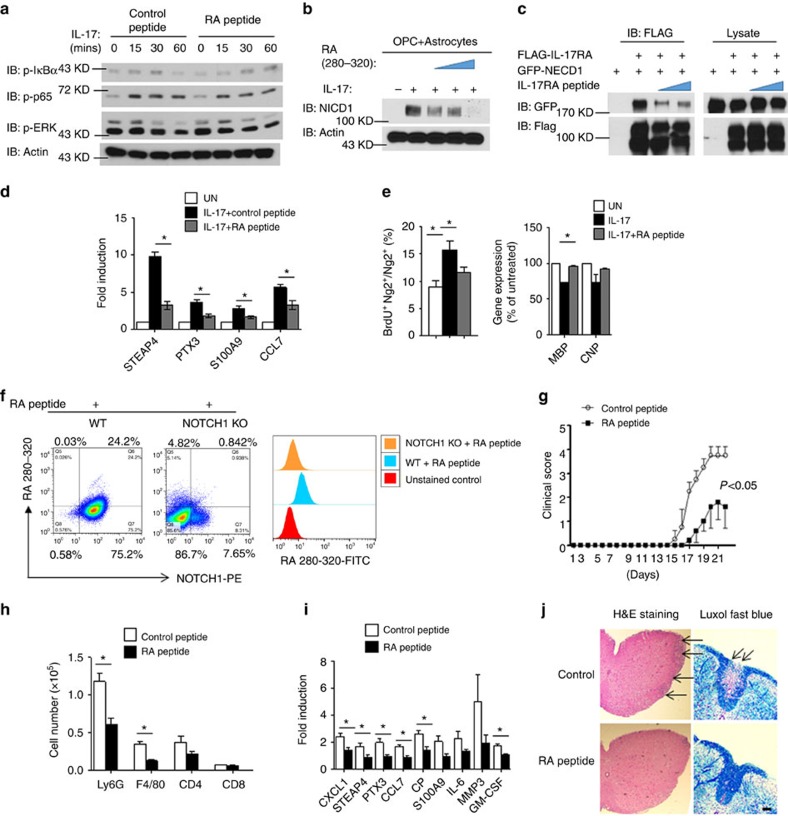
Disrupting IL-17RA–NOTCH1 interaction attenuates Th17-induced EAE. (**a**) Mouse embryonic fibroblasts were pretreated with control peptide or IL-17RA decoy peptide for 2 h (200 μM). Pretreated cells were stimulated with IL-17 (50 ng ml^−1^) for indicated time, followed by immunoblot analysis. (**b**) OPCs co-cultured with Act1 KO astrocytes were stimulated by IL-17 (50 ng ml^−1^) in the presence of different doses of IL-17RA decoy peptide (50,100 and 200 μM), followed by immunoblotting analysis. (**c**) HEK293 cells were transfected with indicated constructs. IL-17RA decoy peptide (100 or 200 μM) was added after transfection. Cell lysates were immunoprecipitated with anti-FLAG antibody, followed by immunoblot analysis. (**d**) OPCs co-cultured with IL-17RA KO astrocytes were pretreated with IL-17RA decoy peptide (200 μM) or left untreated followed by IL-17 stimulation (50 ng ml^−1^). Expressions of indicated genes were analysed by RT–PCR. (**e**) OPCs co-cultured with IL-17RA KO astrocytes were pretreated with RA peptide (200 μM) or left untreated followed by IL-17 (50 ng ml^−1^) stimulation. Treated cells were subjected BrdU incorporation assay. A total of 1,000 NG2^+^ cells were enumerated from 10 different views for BrdU positivity (left panel). Cells receiving the same treatment were analysed for MBP and CNP expression (right panel). (**f**) Wild-type OPCs and NOTCH1 KO OPCs were incubated for FITC-labelled IL-17RA decoy peptide (200 μM) followed by staining with fluorophore (PE)-labelled anti-NOTCH1 antibody. Cells were analysed by flow cytometry for FITC and PE signal. (**g**) Clinical scores of EAE symptoms of mice receiving indicated treatment (*P*<0.001, two-way ANOVA). (**h**) Infiltrating cells from the brain of EAE mice (*n*=5) receiving indicated treatment were analysed by flow cytometry. (**i**) RT–PCR analysis of inflammatory gene expression in spinal cords of mice receiving indicated treatment (*n*=5). (**j**) H&E and LFB staining of sections of spinal cords from mice receiving indicated treatment. Arrow in the H&E staining indicates infiltrated area, and arrow in the LFB staining indicates demyelination area. Scale bar, 100 μm. IB, immunoblotting; IP, immunoprecipitation; WCL, whole-cell lysates. All error bars represent s.e.m. of biological replicates. *P<0.05 based on Mann–Whitney *U*-test. Data are representative of two independent experiments.

Based on surface-exposed regions of the sequence of a protein, a decoy peptide may have the ability to bind to and occupy the docking site of the interacting partner of the original protein, interrupting the protein–protein interaction^38^. Our deletion analysis showed that IL-17RA and NOTCH1 interacted with each other via their extracellular domains, and amino-acid residues 280–320 in IL-17RA were required for the interaction of IL-17RA with NOTCH1 (Fig. 3e). To disrupt the interaction between IL-17RA and NOTCH1, a decoy peptide (with a fluorescein isothiocyanate (FITC) tag at its C terminus) was generated based on the sequence from 280–320 of IL-17RA (RA peptide). As a control, we showed that the RA peptide did not have any impact on IL-17-induced NF-κB and MAPK activation (Fig. 9a), suggesting that this peptide probably does not interrupt IL-17 binding to the receptor. Strikingly, the addition of RA peptide greatly reduced IL-17-induced NOTCH activation (NICD1 cleavage) in OPC co-culture with Act1 KO astrocytes (Fig. 9b). The co-immunoprecipitation experiment showed that this peptide interrupted IL-17RA and NOTCH1 binding (Fig. 9c). Furthermore, the decoy peptide completely abolished IL-17-induced inflammatory gene expression in OPC+astrocyte co-culture (Fig. 9d) and attenuated the IL-17-mediated impact on OPC proliferation and differentiation (Fig. 9e).

To confirm that the RA peptide could directly bind to NOTCH1, we stained wild-type and NOTCH1 KO OPCs with the decoy RA-peptide-FITC and NOTCH1 antibody. Flow cytometry analysis showed that wild-type OPCs, but not NOTCH1 KO OPCs, co-stained with NOTCH1 and RA-peptide-FITC, implicating that this peptide could bind directly to NOTCH1 (Fig. 7f). The residual NOTCH1-positive signalling in the NOTCH1 KO sample might be due to incomplete depletion of NOTCH1 by adenovirus expressing cre-mediated depletion (Fig. 9f). Notably, most of the OPCs were NOTCH1 positive (Fig. 9f), which is consistent with the literature^26^ and the data shown in Fig. 7a. Since this decoy peptide was efficacious for blocking IL-17-induced NOTCH1 activation in co-cultures (without inhibition of TGF-β*-*induced NOTCH1 activation, Supplementary Fig. 8), we next tested whether it had any therapeutic effect on Th17-induced EAE. To overcome the blood–brain barrier, we used an osmotic pump to continuously deliver RA peptide into the CNS by intracerebroventricular injection. Interestingly, the administration of RA peptide significantly attenuated the Th17-mediated EAE severity (Fig. 9g), reduced the CNS-infiltrated macrophages and neutrophils (Fig. 9h) and decreased Th17-induced expression of *NOTCH* genes in the spinal cord (Fig. 9i). Histopathological analysis also indicated reduced inflammatory infiltration and demyelination in spinal cords in the peptide-treated group (Fig. 9j). Thus, the RA peptide has a potent therapeutic effect on Th17-mediated EAE, probably by disrupting the IL-17RA–NOTCH axis in OPCs.

## Discussion

**Figure 10:**
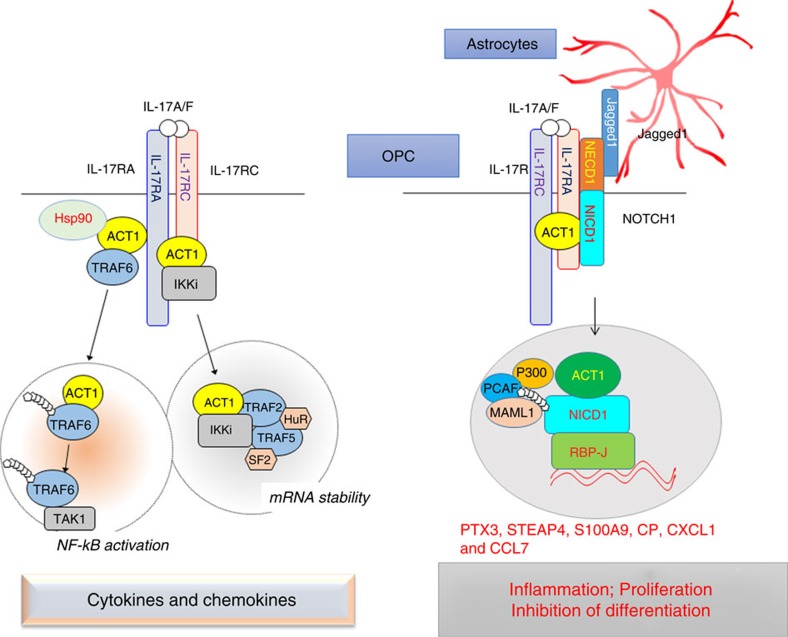
Model of IL-17–NOTCH pathway. Left panel: canonical IL-17 signalling. IL-17 signals through a heterodimeric receptor complex composed of IL-17RA and IL-17RC. On IL-17 stimulation, Act1 is recruited to IL-17R and subsequently engages TRAF6 and Hsp90 to activate NF-κB pathway. In addition, IL-17 stimulation also promotes the formation of Act1–IKKi complex, which in turn engages TRAF2 and TRAF5 to activate the mRNA stabilization pathway. Canonical IL-17 signalling results in transcription of pro-inflammatory and neutrophil-mobilizing cytokines and chemokines; Right panel: IL-17–NOTCH pathway. In the OPC-astrocyte co-culture system, IL-17 stimulation induces NOTCH1 activation in the OPCs, resulting in inflammatory gene expression accompanied by enhanced cell proliferation and impaired maturation. IL-17 receptor A (IL-17RA) can directly interact with the NOTCH receptor NOTCH1, leading to the cleavage of the NICD. Furthermore, Act1, the adaptor protein for IL-17 signalling, forms a complex with NICD in response to IL-17 stimulation. The Act1–NICD complex translocates into the nucleus where Act1–NICD and transcription factor RBP-J are recruited to the promoters of NOTCH target genes important for inflammation and cell proliferation.

In the present study, we report for the first time that IL-17 crosstalks with NOTCH1, a pathway that is known to promote OPC proliferation and suppress OPC differentiation, contributing to demyelinating disease. IL-17R interacts with NOTCH1 via the extracellular domain, which facilitates the cleavage of NICD1, formation of the Act1–NICD1 complex and subsequent translocation into the nucleus. As a result, Act1 and the transcription factor RBP-J are recruited to the promoters of several Th17-induced NOTCH1 target genes, such as *STEAP4*, which are critical for inflammation and cell proliferation. Mechanistically, we found Act1 enhanced the interaction of NICD1 with co-activators of the transcription factor RBP-J, thereby promoting expression of the target genes. Selective genetic ablation of either NOTCH1 or RBP-J in OPCs attenuated the development and severity of Th17- but not Th1-mediated EAE. A decoy peptide of IL-17RA 280–320, which was required for NOTCH–IL-17RA interaction, inhibited IL-17-induced NOTCH1 activation, blocked the impact of IL-17 on the OPC inflammatory response, proliferation and maturation, and attenuated Th17-mediated EAE. Taken together, these findings demonstrated that IL-17-induced NICD1–Act1 nuclear translocation promoted inflammatory gene induction in OPCs that enhanced cell proliferation and interfered with OPC maturation, providing a new mechanism for the IL-17 and NOTCH1 pathways in demyelinating disease (Fig. 10).

Act1 is a key adaptor molecular in IL-17 pathway and plays a very important role in IL-17 signal transduction^17^. Herein, we report for the first time a nucleus function of Act1. On ligand binding, NOTCH1 receptor is cleaved by ADAM family metalloproteases, followed by intramembrane γ-secretase complex, generating NICD that translocates into nucleus to convert the DNA-binding protein RBP-J from a transcriptional repressor into an activator. This process involves the formation of a stable complex which composes of NICD, RBP-J and Mastermind-like family of co-activators (MAML), and the complex serves as a platform to further recruit other co-activators for the activation of NOTCH target genes^20^,^21^. We now found that Act1 forms a complex with NICD–RBP-J and increases the recruitment of co-activators MAML1, P300 and PCAF to NICD–RBP-J. It is possible that in response to IL-17 treatment, Act1 might recruit additional co-activators to NICD–RBP-J, directing NICD–RBP-J binding specifically to the Th17-induced NOTCH target genes, including *STEAP4, PTX3, S100A9* and *CCL7* (Fig. 10).

In the present study, we found that IL-17 could not activate NOTCH1 in OPC single culture conditions, but it could fully activate NOTCH1 in OPCs co-cultured with astrocytes. Jagged1 expressed on astrocytes has been shown to inhibit OPC differentiation and myelination^26^, making it a possible candidate in the IL-17-induced NOTCH1 activation process. Indeed, the co-culture of OPCs with Jagged1 KO astrocytes abolished IL-17-induced NOTCH1 activation, indicating that Jagged1 is indispensable for IL-17-induced NOTCH1 activation. One of the key events in NOTCH activation is the release of the NOTCH ectodomain through ligand-induced and ADAM-mediated NOTCH cleavage at cleavage site S2. This cleavage site resides within the negative regulatory region of NOTCH, which functions to prevent NOTCH activation. Since our data showed that IL-17R interacted with the extracellular domain of NOTCH1 (NECD1), it is possible that the IL-17R–NECD1 interaction may disrupt the inhibitory effect of the negative regulatory region of NOTCH1, facilitating ADAM-mediated NOTCH1 cleavage at cleavage site S2 followed by cleavage at S3 and S4 by the intramembrane γ-secretase complex for NOTCH1 activation. Alternatively, the IL-17R–NECD1 interaction may transduce the signal to the Notch1 transmembrane domain, directly promoting cleavage at S3 and S4 via the intramembrane γ-secretase complex to release NICD1. Future studies are required to elucidate the detailed molecular mechanism by which the IL-17RA–NOTCH1 interaction promotes NOTCH1 activation.

The Th17 and IL-17 pathways play an essential role in many autoimmune diseases including MS^39^,^40^. Specific ablation of Act1, NOTCH1 and RBP-J in OPCs greatly attenuated EAE development, suggesting that OPCs are the major CNS cellular target of the IL-17 and NOTCH pathways in EAE^19^. While IL-17 induced a characteristic inflammatory response in OPCs *ex vivo*, IL-17 also crosstalks with NOTCH1 to upregulate Th17-induced NOTCH1 target genes that are important for both inflammation and cell proliferation. The knowledge that most OPCs are NOTCH1-positive suggests that IL-17RA–NOTCH crosstalk is probably the dominant mode of OPC signalling in response to IL-17 stimulation. In support of this hypothesis, RA peptide that blocks IL-17RA–NOTCH1 interaction inhibited IL-17-induced NOTCH1 activation and Th17-mediated EAE, indicating a critical impact of the IL-17–NOTCH1 axis in Th17–IL-17-dependent EAE pathogenesis. Based on our findings, we propose that the IL-17/Act1/NOTCH axis has dual roles in progenitor cells. While IL-17/NOTCH induces inflammatory gene expression, it also promotes progenitor cell proliferation via some of the target genes such as *STEAP4*. Both processes can be inhibitory to the normal differentiation programme of the progenitor cells (to become mature oligodendrocytes), resulting in a lack of sufficient remyelination (contributing to demyelination). Future studies are required to carefully determine the relative importance of NOTCH1 canonical versus Th17-induced target genes in OPC proliferation/differentiation and consequent demyelinating disease.

## Methods

### Mice

B6.129X1-Notch1^tm2Rko^/GridJ (stock number 006951), Jag1^tm2Grid^/J (stock number 010618) and B6.Cg-Tg (Cspg4-cre/Esr1*) BAkik/J (stock number 008538) were purchased from Jackson Laboratory. RBP-J^*fl*/*fl*^ mice were provided by Dr Tasuku Honjo and Dr Hu, Xiaoyu at Kyoto University and Tsinghua University. LSL–HA–Act1 knock-in mice were generated on a C57BL/6 background by replacing exon 2 of *Traf3ip2* with a LoxP-Stop-LoxP-Traf3ip2 cDNA-HA Tag-PolyA cassette. All the mice used in this study were female. For all experiments, the mice were age-matched (8–12 weeks) littermates between experimental groups. The mice were housed under specific pathogen-free conditions. All animal studies were approved by the Institutional Animal Care and Use Committee of Cleveland Clinic.

### Cells

The HEK293T and HeLa cell lines were obtained from the ATCC and authenticated by analysing the morphology, growth curve and isoenzyme. Cells were tested for mycoplasma contamination and were shown to be mycoplasma negative. Both of these two cell lines were cultured in DMEM containing 10% heat-inactivated FBS (foetal bovine serum, Gibco Cat: 10,438,026) and 1% pen/strep (Thermo Fisher Scientific, Cat: 15,140,122).

### Reagents

For immunoblot analysis, antibodies against NOTCH1, MAML1, PCAF, RBP-J, NICD1, Jagged1, cleaved NOTCH1, rabbit control IgG, cleaved caspase-3, α-tubulin, H3, Myc, p-IκB*α*, p-p65, p-ERK, FLAG, HA and GFP were purchased from Cell Signaling Biotechnology (CST, Cat No. 3,447, 11,959, 3,378, 5,313, 4,147, 2,620, 4,147, 3,900, 9,664, 3,873, 4,499, 2,276, 2,859, 3,033, 4,370, 14,793, 3,724 and 2,956); antibodies against Act1, P300, TRAF6 were purchased from Santa Cruz Biotechnology (sc-11,444, sc-584 and sc-7,221); the antibody against Actin was purchased from Sigma (A5,441); and the antibody against V5 was from Thermo Fisher Scientific (MA5-15,253). All antibodies were used at a dilution of 1:1,000 for western blotting unless otherwise specified. For immunofluorescence, antibodies against FLGA and HA were purchased from CST (14,793) and Sigma (H9,658), respectively; the antibody against NG2 was provided by W.B. Stallcup (Burnham Institute for Medical Research); the antibody against Ki67 was purchased from Abcam (Cat No. ab15,580); the antibody against cleaved NOTCH1 was from Abcam (ab8925); the antibody against GST-π was from CST (3,174) and Enzo (ADI-MSA-102-E); and the antibody against PDGFRα was from eBioscience (14-1,401-81). Antisera against STEAP4, IL-17RA or Act1 was generated by Covance using selected peptides as immunogens. 4,6-Diamidino-2-phenylindole (DAPI) was purchased from Sigma (D5942). All antibodies were used at a dilution of 1:1,000 for western blotting unless otherwise specified.

### Plasmids

Human NOTCH1 and Act1 cDNA were cloned into the pCDNA3.1 (+) vector with FLAG or HA sequences appended to the C terminus of the protein. FLAG–GFP–NOTCH1, GFP–NICD1 and GFP–NECD1 plasmids were kindly provided by Dr Valina L. Dawson at Johns Hopkins University School of Medicine. FLAG–NICD1 was kindly provided by Dr Xiaoxu Hu at Tsinghua University. FLAG and V5 tagged IL-17RA plasmids were generated by cloning human IL-17RA into pCDNA3.1 (+) vector with V5 or FLAG sequence appended to the C terminus. IL-17RA deletion mutants and Act1 deletion mutants were constructed using a Quick Change mutagenesis kit from Agilent (200,518) according to the manufacturer's instruction. The primers used for mutagenesis are listed in Supplementary Table 1.

### Immunoblot and immunoprecipitation

Cells were collected and lysed on ice in lysis buffer containing 0.5% Triton X-100, 20 mM HEPES pH 7.4, 150 mM NaCl, 12.5 mM β-glycerophosphate, 1.5 mM MgCl_2_, 10 mM NaF, 2 mM dithiothreitol, 1 mM sodium orthovanadate, 2 mM EGTA, 20 mM aprotinin and 1 mM phenylmethylsulfonyl fluoride for 20 min, followed by centrifugation at 12,000 r.p.m. for 15 min to extract clear lysates. For immunoprecipitation, the cell lysates were incubated with 1 μg of antibody and A-Sepharose beads at 4 °C overnight. After incubation, the beads were washed four times with lysis buffer, and the precipitates were eluted with 2 × sample buffer. The eluates and whole-cell extracts were resolved by SDS–PAGE followed by immunoblotting with antibodies. Nuclear fractionation was performed using the NUCLEI EZ PREP kit purchased from Sigma in accordance with the manufacturer's instructions. For cell fractionation, we used the NUCLEI EZ PREP NUCLEI ISOLATION KIT from Sigma (Cat No. NUC-101) to isolate nuclear and cytoplasmic proteins. Densitometric quantification of the western blot results was performed on images of scanned films using ImageJ software. All uncropped western blots can be found in Supplementary Fig. 9.

### Immunofluorescence

Transfected cells or frozen sections were fixed with 4% paraformaldehyde (PFA) followed by permeabilization with PBS containing 0.3% Triton X-100 for 10 min. Before incubation with primary antibody, the samples were incubated with 10% goat serum at room temperature for 1 h to block non-specific staining. After 12 h of incubation with primary antibody at 4 °C, the samples were washed three times with ice-cold PBS and further stained with fluorophore (Alex488 and Alexa 530)-conjugated secondary antibodies. After staining, the samples were counterstained with DAPI and immersed in mounting medium before being sealed on a slide with nail polish. The sealed slides were analysed using a Leica TCS-SP microscope with companion software. Quantification of immunofluorescent staining was performed by manually enumerating the number of positive cells in 20 views per biological or technical replicate.

### Mass spectrometry identification

Samples were reduced and alkylated in dithiothreitol and iodoacetamide followed by trypsin digestion overnight. Digested samples were injected onto an Agilent Zorbax 300SB-C18 0.075 mm × 150 mm column on an Eskigent nanoLC system coupled to a Thermo LTQ-ETD-Orbitrap. The Advion Triversa NanoMate served as the nano-ion spray source. Mass spectrometry and tandem mass spectrometry (MSMS) data were searched against the RefSeq human protein database by Sorcerer Sequest (Sage-N Research, Milpitas, CA). The searched data set was processed using the TPP (trans-proteomics pipeline) and filtered with peptide prophet.

### PLA

PLA was performed using the Duolink In Situ Red Kit purchased from Sigma (DUO92101) in accordance with the manufacturer's instruction. Briefly, transfected cells were washed once with ice-cold PBS, followed by fixation with 4% PFA for 15 min at room temperature. The fixed cells were then washed three times with PBS and permeabilized with 0.3% Triton X-100 containing PBS for 10 min. The permeabilized cells were blocked with 5% normal goat serum for 1 h at room temperature. The cells were then incubated with primary antibodies (rabbit anti-FLAG (CST 14793, 1:300 dilution) and mouse anti-HA (Sigma H9658, 1:300 dilution) diluted in 10% normal goat serum supplemented with 0.1% Tween at 4 °C overnight. Following the incubation, the cells were three times with PBS and then incubated with two PLA probes (Duolink In Situ PLA Probes Anti-rabbit PLUS and Anti-Mouse MINUS, Sigma-Aldrich) for 1 h at 37 °C. After probe incubation, the samples were incubated in ligation solution for 1 h at 37 °C. After ligation, the cells were washed with wash buffer A and incubated in amplification solution for 2 h at 37 °C. The cells were then serially washed twice in 1 × wash buffer B, 0.01 × wash buffer B once, and PBS once, followed by incubation with secondary antibodies for 1 h at room temperature. Finally, the cells were washed three times with PBS and mounted in Duolink In Situ Mounting Medium supplemented with DAPI. Fluorescent images were obtained using a confocal microscope.

### Primary cell culture

The astrocyte culture was prepared from 1-day-old neonatal mice. Brains freed of meninges were dissociated with 1-ml pipettes. Debris was removed by filtration through a 70-μm cell strainer (Falcon). The cells were cultured in DMEM plus 10% foetal bovine serum (vol/vol) supplemented with 50 μg ml^−1^ penicillin and 50 μg ml^−1^ streptomycin. Ten days after the initial culture, cells were stained with antibody against GFAP (Sigma, G3893, 1:500) to verify the purity of the astrocytes. OPCs were derived from neurospheres. Embryos were dissected from pregnant mice 14 days after the last recording of a vaginal plug. Neurospheres were cultured in DMEM/F12 supplemented with B27 (Invitrogen) and 20 ng ml^−1^ recombinant mouse epidermal growth factor (R&D). Floating neurospheres were passaged at a 1:3 ratio in the same medium every 3 days. To produce pure OPCs, neurospheres were dissociated after two–three passages and plated on poly-D-lysine-coated plates in DMEM/F12 supplemented with B27 (Invitrogen), 20 ng ml^−1^ FGF and 10 ng ml^−1^ PDGF (Peprotech). The OPC purity was verified with anti-Olig2 and CNPase staining (>98% of cells were Olig2^+^ CNPase^−^ at 4 days after culture). For the OPC-astrocyte co-culture, astrocytes were plated in six-well plates at a density of 2 × 10^4^ per well in DMEM/F12 supplemented with B27. On the following day, dissociated neurospheres were plated together with the astrocytes at a density of 5 × 10^5^ per well. The culture medium was further supplemented with 20 ng ml^−1^ FGF+10 ng ml^−1^ PDGF to promote OPC differentiation. The co-culture was maintained for 4 more days before being subjected to experiments (for example, IL-17 treatment). To generate NOTCH1 and RBP-J knockout OPCs, neurospheres were prepared from NOTCH1^*fl*/*fl*^ and RBP-J ^*fl*/*fl*^ embryos. An adenovirus directing the expression of GFP (control) or Cre was added to the culture medium after neurosphere dissociation to mediate the deletion of floxed alleles. NOTCH1 and Jagged1-deficient astrocytes were isolated from NOTCH1 ^*fl*/*fl*^ and Jagged1 ^*fl*/*fl*^ neonatal mice, and deletion was achieved by adenovirus infection as described for the neurosphere. The Cre deletion was validated in OPCs for NG2-ER-cre. Cultured OPCs were collected and stained with anti-NG2 for cell sorting of NG2^+^ OPCs cells. Sorted cells were lysed, and western blotting was conducted to evaluate RBP-J expression in NG2^+^ cells from NG2-ER-cre; RBP-J ^*fl*/+^ and NG2-ER-cre; RBP-J ^*fl*/*fl*^ mice.

### Real-time PCR

Total RNA was extracted from the spinal cord with TRIzol (Invitrogen) according to the manufacturer's instructions. Three micrograms of total RNA for each sample was reverse-transcribed using SuperScript II Reverse Transcriptase from Thermo Fisher Scientific. The resulting complementary DNA was analysed by real-time polymerase chain reaction (PCR) using SYBR Green Real-Time PCR Master Mix. All gene expression results are expressed as arbitrary units relative to the expression of *Actb*. The fold induction of gene expression in spinal cord after EAE induction was determined by dividing the relative abundance of experimental samples by the mean relative abundance of control samples from naive mice. The primer sequences for RT–PCR are listed in Supplementary Table 1.

### Luciferase reporter assay

HeLa cells were transfected with 100 ng of a Hes1-luciferase reporter construct in a 12-well plate together with the combination of plasmids indicated in the figure legends. Forty-eight hours after transfection, the cells were lysed, and luciferase activity was determined using the luciferase assay system and chemiluminescent reagents from Promega.

### Chromosomal immunoprecipitation

The binding of Act1 and RBP-J to promoters of STEAP4, PTX3, CCL7 and S100A9 was assessed by chromosomal immunoprecipitation (ChIP) using OPCs co-cultured with Act1 KO astrocytes. The ChIP assay was performed using the ChIP assay Kit from Active Motif in accordance with the manufacturer's instructions. Briefly, a total of 1 × 10^7^ co-cultured cells was fixed with 1% formaldehyde. Fixed cells were sonicated for 30 s at medium power for 20 cycles, followed by centrifugation. The sonicated whole-cell extract was diluted in ChIP dilution buffer. The samples were incubated at 4 °C overnight with antibodies against RBP-J (CST 5313, diluted at 1:50), Act1 (Covance, diluted at 1:50) or rabbit IgG (CST Cat No. 3,900, at 1:50) and A-Sepharose beads (Active Motif). The precipitates were washed and subjected to DNA extraction followed by RT–PCR analysis of promoter enrichment using the primers listed in Supplementary Table 1.

Mouse control primers for the Chip assay were purchased from Active Motif (71011).

### Tamoxifen injection to deplete NOTCH1 and RBP-J *in vivo*

NG2-ER-cre; NOTCH1 ^*fl*/+^ and NG2-ER-cre; NOTCH1^*fl*/*fl*^ mice and NG2-ER-cre; RBP-J ^*fl*/+^ and NG2-ER-cre; RBP-J ^*fl*/*fl*^ mice (4 weeks old) were injected intraperitoneally with 5 mg of tamoxifen per week for at least 4 weeks.

### GeneChip Microarray analysis

Five mice in each group were left untreated (naive group) or adoptively transferred with MOG-reactive Th1 or Th17 cells. Act1 KO mice received only the Th17 adoptive transfer. Twelve days after adoptive transfer, the mice were killed, and spinal cords were subjected to mRNA extraction for microarray analysis. Target preparation was performed on a Biomek FXP (Beckman Coulter, Brea, CA) using a GeneChip HT 3′IVT Express Kit (Affymetrix, Santa Clara, CA) in accordance with the manufacturer's instruction. Labelled cRNA was hybridized on an Affymetrix GeneChip HT-MG-430PM-96 (Affymetrix). Array hybridization, washing and scanning were performed using GeneTitan (Affymetrix). Three independent biological replicates were analysed in each experiment, which yielded consistent results. The *t*-test was used to assess significance, and *P*<0.05 was deemed significant. The data for the normalized GeneChip analysis are provided in Supplementary Data 1 and the raw data are deposited in the Gene Expression Omnibus.

### Induction and assessment of EAE

Recipient mice were injected with 3.0 × 10^7^ MOG_35–55_-reactive Th1 or Th17 cells at 4 h after 500-Rad sublethal irradiation. To prepare MOG_35–55_-reactive T cells, donor mice were immunized with MOG_35−55_ subcutaneously; draining lymph node cells and splenocytes were prepared from donor mice at 10 days after immunization. The cells were cultured for 5 days with MOG_35–55_ at a concentration of 25 μg ml^−1^ under either Th1-polarizing conditions (20 ng ml^−1^) rmIL-12, R&D; 2 μg ml^−1^ αIL-23p19, eBioscience) or Th17-polarizing conditions (20 ng ml^−1^ rmIL-23, R&D). Scoring of EAE symptoms was performed in a double-blinded manner. Power analysis was performed to determine the sample size before the experiment. With an average coefficient of variance of 25%, we determined that *n*=5 was needed to obtain 90% power for the detection of a 30% difference between the groups. The mice were weighed and assigned scores daily for neurological signs according to the following scale: 0, no disease; 1, decreased tail tone or slightly clumsy gait; 2, tail atony and/or moderately clumsy gait and/or poor slighting ability; 3, limb weakness; 4, limb paralysis; 5, moribund state or death. The control and experimental groups were blinded to the investigators who recorded the clinical score, flow analysis, histology and Luxol fast blue (LFB) staining.

### Isolation and analysis of CNS inflammatory cells

Brains were homogenized in ice-cold tissue grinders and filtered through a 70-μm cell strainer, and the cells were collected by centrifugation at 400*g* for 5 min at 4 °C. The cells were re-suspended in 10 ml of 30% Percoll (Amersham Bioscience) and centrifuged onto a 70% Percoll cushion in 15-ml tubes at 800*g* for 30 min. The cells in the 30–70% interface were collected and subjected to flow cytometry. The gross cell population was first gated based on the forward and side scatter for viable, single cell events. Fluorescence-conjugated monoclonal antibodies against CD4 (clone GK1.5), CD8 (clone 53-6.7), CD45 (clone 30-F11), Ly6G (clone 1A8), CD19 (clone 1D3) and iso-type controls were purchased from BD Biosciences. F4/80 (clone Cl:A3-1) was obtained from Serotech. The antibodies were diluted 1:200–1:400 for use.

### Histological analysis

All sections used herein were 5-μm thick. For paraffin-embedded tissue, spinal cords collected from phosphate-buffered saline-perfused mice were fixed in 10% formalin and then dehydrated with 70% alcohol. The sections were stained with either haematoxylin and eosin (H&E) or LFB to evaluate inflammation and demyelination, respectively. For cytohistochemical staining, the cells were fixed in 2% PFA for 10 min. The cells were further permeabilized with 0.1% Triton X-100 for 10 min and then incubated with primary antibody followed by fluorescence-conjugated secondary antibodies for microscopic analysis. NG2 antibodies were provided by W.B. Stallcup (Burnham Institute for Medical Research). Histological quantification was performed in a double-blinded manner.

### Peptide

Scramble and IL-17RA decoy peptides were synthesized as described^38^. The scramble peptide sequence is ‘PRFASPCCVFCTPDVPQSYTNVVLLITIHVVDQPAKSNVS', and the decoy peptide sequence is ‘VQVQPFFSSCLNDCLRHAVTVPCPVISNTTVPKPVADYIP'.

### Osmotic pump implantation

Control or IL-17RA decoy peptide was dissolved in sterile artificial cerebrospinal fluid (CSF). Micro-osmotic pumps (Alzet) were filled with dissolved peptides and placed in the bottom of a 200-ml glass beaker that was half filled with sterile 0.9% NaCl in a humidified 37 °C incubator overnight for activation. Mice were anaesthetized with a ketamine (100 mg kg^−1^) and xylazine (10 mg kg^−1^) cocktail. Anaesthetized mice were placed in a stereotactic device with a three-axis chronic micromanipulator. The injection coordinates were 1 mm lateral, 0.3 mm posterior and 2 mm deep to the bregma. The overlying skin was cut to form a 0.7 cm incision in the head and a 0.5 cm incision in the back of the mouse right below the neck. The cranium was opened using a compact drill followed by insertion of the infusion cannula (2 mm in depth). Osmotic pumps were implanted in the back of the mice. A pocket was made to connect the infusion pump so that the incision site was not directly over the pump. Implanted mice were housed in separate cages to avoid fighting. Three days after pump implantation, Th17 cells were adoptively transferred into the mice by intraperitoneal injection.

### Statistics

For comparison between two groups, normality was not assessed for the statistical analysis. Non-parametric statistics were applied to compare differences between two groups. The Mann–Whitney *U*-test was used to derive all *P* values. The clinical scores in the EAE experiment were analysed with two-way ANOVA for multiple comparisons. The sample size for the EAE experiment was estimated by power analysis assuming a normal distribution of the data. No randomization was performed. *P*<0.05 was considered significant. The results are presented as the mean, and the error bars represent the s.e.m. technical or biological replicates as indicated in the figure legend.

### Data availability

All data supporting the findings of this study are available within the article and its Supplementary Information files or from the corresponding authors on reasonable request. The GeneChip microarray data described in the study have been deposited in the Gene Expression Omnibus under accession code GSE97035.

## Additional information

**How to cite this article:** Wang, C. *et al*. IL-17 induced NOTCH1 activation in oligodendrocyte progenitor cells enhances proliferation and inflammatory gene expression. *Nat. Commun.* **8**, 15508 doi: 10.1038/ncomms15508 (2017).

**Publisher's note:** Springer Nature remains neutral with regard to jurisdictional claims in published maps and institutional affiliations.

## Supplementary Material

Supplementary Information Supplementary Figures, Supplementary Tables.

Supplementary Data 1 Microarray analysis of the gene expression in spinal cords of Th1- and Th17- induced EAE mice.
