# Supplementary material for: IL-17 induced NOTCH1 activation in oligodendrocyte progenitor cells enhances proliferation and inflammatory gene expression
Source: Nat Commun. 2017 May 31;8:15508. doi: 10.1038/ncomms15508 (PMC5460031; doi:10.1038/ncomms15508)
Supplement: Supplementary Information — Supplementary Figures, Supplementary Tables. [file ncomms15508-s1.pdf]

Supplementary Information

Supplementary Figure 1.

NOTCH1

>gi|27894368|ref|NP_060087.2| notch1 preproprotein [Homo sapiens]

MPPLLAPLLC LALLPALAAR GPRCSQPGET CLNGGKCEAA NGTEACVCGG AFVGPRCQDP NPCLSTPCKN AGTCHVVDRR GVADYACSCA LGFSGPLCLT PLDNACLTNP CRNGGTCDLL
TLTEYKCRCP PGWSGKSCQQ ADPCASNPCA NGGQCLPFEA SYICHCPPSF HGPTCR**QDVN ECGQKPGLCR** HGGTCHNEVG SYRCVCRATH TGPNCERPYV PCSPSPCQNG GTCRPTGDVT
HECACLPGFT GQNCEENIDD CPGNNCKNGG ACVDGVNTYN CRCPPEWTGQ YCTEDVDECQ LMPNACQNGG TCHNTHGGYN CVCVNGWTGE DCSENIDDCA SAACFHGATC HDR**VASFYCE**
CPHGRTGLLC HLNDACISNP CNEGSNCDTN PVNGKAICTC PSGYTGPACS QDVDECSLGA NPCEHAGKCI NTLGSFECQC LQGYTGPRCE IDVNECVSNP CQNDATCLDQ IGEFQCICMP
GYEGVHCEVN TDECASSPCL HNGRCLDKIN EFQCECPTGF TGHLCQYDVD ECASTPCKNG AKCLDGPNTY TCVCTEGYTG THCEVDIDEC DPDPCHYGSC KDGVATFTCL CRPGYTGHHC
ETNINECSSQ PCRHGGTCQD RDNAYLCFCL KGTTGPNCEI NLDDCASSPC DSGTCLDKID GYECACEPGY TGSMCNINID ECAGNPCHNG GTCEDGINGF TCRCPEGYHD PTCLSEVNEC
NSNPCVHGAC RDSLNGYKCD CDPGWSGTNC DINNNECESN PCVNGGTCKD MTSGYVCTCR EGFSGPNCQT NINECASNPC LNQGTCIDDV AGYKCNCLLP YTGATCEVVL APCAPSPCRN
GGECRQSEDY ESFSCVCPTG WQAGQTCEVD INECVLSPCR HGASCQNTHG GYRCHCQAGY SGRNCETDID DCRPNPCHNG GSCTDGINTA FCDCLPGFRG TFCEEDINEC ASDPCRNGAN
CTDCVDSYTC TCPAGFSGIH CENNTPDCTE SSCFNGGTCV DGINSFTCLC PPGFTGSYCQ HDVNECDSQP CLHGGTCQDG CGSYRCTCPQ GYTGPNCQNL VHWCDSSPCK NGGKCWQTHT
QYRCECPSGW TGLYCDVPSV SCEVAAQRQG VDVAR**LCQHG GLCVDAGNTH HCR**CQAGYTG SYCEDLVDEC SPSPCQNGAT CTDYLGGYSC KCVAGYHGVN CSEEIDECLS HPCQNGGTCL
DLPNTYKCSC PR**GTQGVHCE INVDDCNPPV DPVSR**SPKCF NNGTCVDQVG GYSCTCPPGF VGERCEGDVN ECLSNPCDAR GTQNCVQR**VN DFHCECR**AGH TGRRCESVIN GCKGKPCKNG
GTCAVASNTA RGFICKCPAG FEGATCENDA RTCGSLRCLN GGTCISGPRS PTCLCLGPFT GPECQFPASS PCLGGNPCYN QGTCEPTSES PFYRCLCPAK FNGLLCHILD YSFGGGAGRD
IPPPLIEEAC ELPECQEDAG NKVCSLQCNN HACGWDGGDC SLNFNDPWKN CTQSLQCWKY FSDGHCDSQC NSAGCLFDGF DCQRAEGQCN PLYDQYCKDH FSDGHCDQGC NSAECEWDGL
DCAEHVPERL AAGTLVVVVL MPPEQLRNSS FHFLRELSR**V LHTNVVFK**RD AHGQQMIFPY YGREEELRKH PIKRAAEGWA APDALLGQVK **ASLLPGGSEG GR**RRRELDPM DVRGSIVYLE
IDNRQCVQAS SQCFQSATDV AAFLGALASL GSLNIPYKIE AVQSETVEPP PPAQLHFMYV AAAAFVLLFF VGCGVLLSRK RRRQHGQLWF PEGFKVSEAS KKKRREPLGE DSVGLKPLKN
ASDGALMDDN QNEWGDEDLE TKKFRFEEPV VLPDLDDQTD HRQWTQQHLD AADLRMSAMA PTPPQGEVDA DCMDVNVRGP DGFTPLMIAS CSGGGLETGN SEEEDAPAV ISDFIYQGAS
LHNQTDR**TGE TALHLAAR**YS RSDAAKRLLE ASADANIQDN MGRTPLHAAV SADAQGVFQI LIRNRATDLD ARMHDGTTPL ILAARLAVEG MLEDLINSHA DVNAVDDLGK SALHWAAAVN
NVDAAVVLLK NGANKDMQNN REETPLFLAA REGSYETAK**V LLDHFANR**DI TDHMDRLPRD IAQERMHHDI VRLLDEYNLV RSPQLHGAPL GGTPTLSPPL CSPNGYLGSL KPGVQGKKVR
KPSSKGLACG SKEAKDLKAR RKKSQDGKGC LLDSSGMLSP VDSLESPHGY LSDVASPPLL PSPFQQSPSV PLNHLPGMPD THLGIGHLNV AAKPEMAALG GGGR**LAFETG PPR**LSHLPVA
SGTSTVLGSS SGGALNFTVG GSTSLNGQCE WLSRLQSGMV PNQYNPLRGS VAPGPLSTQA PSLQHGMVGP LHSSLAASAL SQMMSYQGLP STRLATQPHL VQTQQVQPQN LQMQQQNLQP
ANIQQQQSLQ PPPPPPQPHL GVSSAASGHL GRSFLSGEPS QADVQPLGPS SLAVHTILPQ ESPALPTSLP SSLVPPVTAA QFLTPPSQHS YSSPVDNTPS HQLQVPEHPF LTPSPESPDQ
WSSSSPHSNV SDWSEGVSSP PTSMQSQIAR IPEAFK

MONO MW: **272394**, pI: **4.95**
Database = **/home/sorcerer/fasta/RefSeqHuman_082508.fasta**
Links: Google Google Scholar NCBI UniProtKB

Position	Mass	Peptide
177-190	1545.7	QDVNECGQKPGLCR
354-365	1367.58	VASFYCECPHGR
1116-1133	1919.83	LCQHGGLCVDAGNTHHCR
1213-1235	2450.1	GTQGVHCEINVDDCNPPVDPVSR
1289-1297	1121.44	VNDFHCECR
1600-1608	1055.61	VLHTNVVFK
1651-1662	1099.56	ASLLPGGSEGGR
1928-1938	1138.61	TGETALHLAAR
2080-2088	1083.58	VLLDHFANR
2265-2273	986.52	LAFETGPPR

NOTCH2

```
>gi|24041035|ref|NP_077719.2| notch 2 preproprotein [Homo sapiens]
MPALRPALLW ALLALWLCCA APAHALQCRD GYEPCVNEGM CVTYHNGTGY CKCPEGFLGE YCQHRDPCEK NRCQNGGTCV AQAMLGKATC RCASGFTGED CQYSTSHPCF VSRPCLNGGT
CHMLSRDTYE CTCQVGFTGK ECQWTDACLS HPCANGSTCT TVANQFSCKC LTGFTGQKCE TDVNECDIPG HCQHGGTCLN LPGSYQCQCP QGFTGQYCDS LYVPCAPSPC VNGGTCRQTG
DFTFECNCLP GFEGSTCERN IDDCPNHRCQ NGGVCVDGVN TYNCRCPPQW TGQFCTEDVD ECLLQPNACQ NGGTCANRNG GYGCVCVNGW SGDDCSENID DCAFASCTPG STCIDRVASF
SCMCPEGKAG LLCHLDDACI SNPCHKGALC DTNPLNGQYI CTCPQGYKGA DCTEDVDECA MANSNPCEHA GKCVNTDGAF HCECLKGYAG PRCEMDINEC HSDPCQNDAT CLDKIGGFTC
LCMPGFKGVH CELEINECQS NPCVNNGQCV DKVNRFQCLC PPGFTGPVCQ IDIDDCSSTP CLNGAKCIDH PNGYECQCAT GFTGVLCEEN IDNCDPDPCH HGQCQDGIDS YTCICNPGYM
GAICSDQIDE CYSSPCLNDG RCIDLVNGYQ CNCQPGTSGV NCEINFDDCA SNPCIHGICM DGINRYSCVC SPGFTGQRCN IDIDECASNP CRKGATCING VNGFRCICPE GPHHPSCYSQ
VNECLSNPCI HGNCTGGLSG YKCLCDAGWV GINCEVDKNE CLSNPCQNGG TCDNLVNGYR CTCKKGFKGY NCQVNIDECA SNPCLNQGTC FDDISGYTCH CVLPYTGKNC QTVLAPCSPN
PCENAAVCKE SPNFESYTCL CAPGWQGQRC TIDIDECISK PCMNHGLCHN TQGSYMCECP PGFSGMDCEE DIDDCLANPC QNGGSCMDGV NTFSCLCLPG FTGDKCQTDM NECLSEPCKN
GGTCSDYVNS YTCKCQAGFD GVHCENNINE CTESSCFNGG TCVDGINSFS CLCPVGFTGS FCLHEINECS SHPCLNEGTC VDGLGTYRCS CPLGYTGKNC QTLVNLCSRS PCKNKGTCVQ
KKAESQCLCP SGWAGAYCDV PNVSCDIAAS RRGVLVEHLC QHSGVCINAG NTHYCQCPLG YTGSYCEEQL DECASNPCQH GATCSDFIGG YRCECVPGYQ GVNCEYEVDE CQNQPCQNGG
TCIDLVNHFK CSCPPGTRGL LCEENIDDCA RGPHCLNGGQ CMDRIGGYSC RCLPGFAGER CEGDINECLS NPCSSEGSLD CIQLTNDYLC VCRSAFTGRH CETFVDVCPQ MPCLNGGTCA
VASNMPDGFI CRCPPGFSGA RCQSSCGQVK CRKGEQCVHT ASGPRCFCPS PRDCESGCAS SPCQHGGSCH PQRQPPYYSC QCAPPFSGSR CELYTAPPST PPATCLSQYC ADKARDGVCD
EACNSHACQW DGGDCSLTME NPWANCSSPL PCWDYINNQC DELCNTVECL FDNFECQGNS KTCKYDKYCA DHFKDNHCDQ GCNSEECGWD GLDCAADQPE NLAEGTLVIV VLMPPEQLLQ
DARSFLRALG TLLHTNLRIK RDSQGELMVY PYYGEKSAAM KKQRMTRRSL PGEQEQEVAG SKVFLEIDNR QCVQDSDHCF KNTDAAAALL ASHAIQGTLS YPLVSVVSES LTPERTQLLY
LLAVAVVIIL FIILLGVIMA KRKRKHGSLW LPEGFTLRRD ASNHKRREPV GQDAVGLKNL SVQVSEANLI GTGTSEHWVD DEGPQPKKVK AEDEALLSEE DDPIDRRPWT QQHLEAADIR
RTPSLALTPP QAEQEVDVLD VNVRGPDGCT PLMLASLRGG SSDLSDEDED AEDSSANIIT DLVYQGASLQ AQTDRTGEMA LHLAARYSRA DAAKRLLDAG ADANAQDNMG RCPLHAAVAA
DAQGVFQILI RNRVTDLDAR MNDGTTPLIL AARLAVEGMV AELINCQADV NAVDDHGKSA LHWAAAVNNV EATLLLLKNG ANRDMQDNKE ETPLFLAARE GSYEAAKILL DHFANRDITD
HMDRLPRDVA RDRMHHDIVR LLDEYNVTPS PPGTVLTSAL SPVICGPNRS FLSLKHTPMG KKSRRPSAKS TMPTSLPNLA KEAKDAKGSR RKKSLSEKVQ LSESSVTLSP VDSLESPHTY
VSDTTSSPMI TSPGILQASP NPMLATAAPP APVHAQHALS FSNLHEMQPL AHGASTVLPS VSQLLSHHHI VSPGSGSAGS LSRLHPVPVP ADWMNRMEVN ETQYNEMFGM VLAPAEGTHP
GIAPQSRPPE GKHITTPREP LPPIVTFQLI PKGSIAQPAG APQPQSTCPP AVAGPLPTMY QIPEMARLPS VAFPTAMMPQ QDGQVAQTIL PAYHPFPASV GKYPTPPSQH SYASSNAAER
TPSHSGHLQG EHPYLTPSPE SPDQWSSSSP HSASDWSDVT TSPTPGGAGG GQRGPGTHMS EPPHNNMQVY A
```

MONO MW: **265226**, pI: **4.95**

Database = **/home/sorcerer/fasta/RefSeqHuman_082508.fasta**

Links: Google Google Scholar NCBI UniProtKB

Position	Mass	Peptide
53-65	1537.64	CPEGFLGEYCQHR
53-70	2109.87	CPEGFLGEYCQHRDPCEK
127-140	1550.64	DTYECTCQVGFTGK
260-268	1082.46	NIDDCPNHR
357-368	1257.52	VASFSCMCPEGK
409-432	2465.92	GADCTEDVDECAMANSNPCEHAGK
475-487	1372.63	IGGFTCLCMPGFK
666-678	1403.6	YSCVCSPGFTGQR
829-849	2160.94	NCQTVLAPCSPNPCENAAVCK
1049-1058	1027.45	CSCPLGYTGK
1059-1069	1249.59	NCQTLVNLCSR
1082-1111	3100.35	KAESQCLCPSGWAGAYCDVPNVSCDIAASR
1211-1218	819.34	CSCPPGTR
1219-1231	1449.62	GLLCEENIDDCAR
1245-1251	754.34	IGGYSCR
1333-1341	890.41	CPPGFSGAR
1353-1365	1368.66	KGEQCVHTASGPR
1366-1372	808.34	CFCPSPR
1394-1410	1886.81	QPPYYSCQCAPPFSGSR
1568-1578	1207.7	ALGTLLHTNLR
1582-1596	1777.79	DSQGELMVYPYYGEK
1726-1738	1423.79	RREPVGQDAVGLK

NOTCH3

```
>gi|134244285|ref|NP_000426.2| Notch homolog 3 [Homo sapiens]
MGPGARGRRR RRRPMSPPPP PPPVRALPLL LLLAGPGAAA PPCLDGSPCA NGGRCTQLPS REAACLCPPG WVGERCQLED PCHSGPCAGR GVCQSSVVAG TARFSCRCPR GFRGPDCSLP
DPCLSSPCAH GARCSVGPDG RFLCSCPPGY QGRSCRSDVD ECRVGEPCRH GGTCLNTPGS FRCQCPAGYT GPLCENPAVP CAPSPCRNGG TCRQSGDLTY DCACLPGFEG QNCEVNVDDC
PGHRCLNGGT CVDGVNTYNC QCPPEWTGQF CTEDVDECQL QPNACHNGGT CFNTLGGHSC VCVNGWTGES CSQNIDDCAT AVCFHGATCH DRVASFYCAC PMGKTGLLCH LDDACVSNPC
HEDAICDTNP VNGRAICTCP PGFTGGACDQ DVDECSIGAN PCEHLGRCVN TQGSFLCQCG RGYTGPRCET DVNECLSGPC RNQATCLDRI GQFTCICMAG FTGTYCEVDI DECQSSPCVN
GGVCKDRVNG FSCTCPSGFS GSTCQLDVDE CASTPCRNGA KCVDQPDGYE CRCAEGFEGT LCDRNVDDCS PDPCHHGRCV DGIASFSCAC APGYTGTRCE SQVDECRSQP CRHGGKCLDL
VDKYLCRCPS GTTGVNCEVN IDDCASNPCT FGVCRDGINR YDCVCQPGFT GPLCNVEINE CASSPCGEGG SCVDGENGFR CLCPPGSLPP LCLPPSHPCA HEPCSHGICY DAPGGFRCVC
EPGWSGPRCS QSLARDACES QPCRAGGTCS SDGMGFHCTC PPGVQGRQCE LLSPCTPNPC EHGGRCESAP GQLPVCSCPQ GWQGPRCQQD VDECAGPAPC GPHGICTNLA GSFSCTCHGG
YTGPSCDQDI NDCDPNPCLN GGSCQDGVGS FSCSCLPGFA GPRCARDVDE CLSNPCGPGT CTDHVASFTC TCPPGYGGFH CEQDLPDCSP SSCFNGGTCV DGVNSFSCLC RPGYTGAHCQ
HEADPCLSRP CLHGGVCSAA HPGFRCTCLE SFTGPQCQTL VDWCSRQPCQ NGGRCVQTGA YCLCPPGWSG RLCDIRSLPC REAAAQIGVR LEQLCQAGGQ CVDEDSSHYC VCPEGRTGSH
CEQEVDPCLA QPCQHGGTCR GYMGGYMCEC LPGYNGDNCE DDVDECASQP CQHGGSCIDL VARYLCSCPP GTLGVLCEIN EDDCGPGPPL DSGPRCLHNG TCVDLVGGFR CTCPPGYTGL
RCEADINECR SGACHAAHTR DCLQDPGGGF RCLCHAGFSG PRCQTVLSPC ESQPCQHGGQ CRPSPGPGGG LTFTCHCAQP FWGPRCERVA RSCRELQCPV GVPCQQTPRG PRCACPPGLS
GPSCRSFPGS PPGASNASCA AAPCLHGGSC RPAPLAPFFR CACAQGWTGP RCEAPAAAPE VSEEPRCPRA ACQAKRGDQR CDRECNSPGC GWDGGDCSLS VGDPWRQCEA LQCWRLFNNS
RCDPACSSPA CLYDNFDCHA GGRERTCNPV YEKYCADHFA DGRCDQGCNT EECGWDGLDC ASEVPALLAR GVLVLTVLLP PEELLRSSAD FLQRLSAILR TSLRFRLDAH GQAMVFPYHR
PSPGSEPRAR RELAPEVIGS VVMLEIDNRL CLQSPENDHC FPDAQSAADY LGALSAVERL DFPYPLRDVR GEPLEPPEPS VPLLPLLVAG AVLLLVILVL GVMVARRKRE HSTLWFPEGF
SLHKDVASGH KGRREPVGQD ALGMKNMAKG ESLMGEVATD WMDTECPEAK RLKVEEPGMG AEEAVDCRQW TQHHLVAADI RVAPAMALTP PQGDADADGM DVNVRGPDGF TPLMLASFCG
GALEPMPTEE DEADDTSASI ISDLICQGAQ LGARTDRTGE TALHLAARYA RADAAKRLLD AGADTNAQDH SGRTPLHTAV TADAQGVFQI LIRNRSTDLD ARMADGSTAL ILAARLAVEG
MVEELIASHA DVNAVDELGK SALHWAAAVN NVEATLALLK NGANKDMQDS KEETPLFLAA REGSYEAAKL LLDHFANREI TDHLDRLPRD VAQERLHQDI VRLLDQPSGP RSPPGPHGLG
PLLCPPGAFL PGLKAAQSGS KKSRRPPGKA GLGPQGPRGR GKKLTLACPG PLADSSVTLS PVDSLDSPRP FGGPPASPGG FPLEGPYAAA TATAVSLAQL GGPGRAGLGR QPPGGCVLSL
GLLNPVAVPL DWARLPPPAP PGPSFLLPLA PGPQLLNPGT PVSPQERPPP YLAVPGHGEE YPAAGAHSSP PKARFLRVPS EHPYLTPSPE SPEHWASPSP PSLSDWSEST PSPATATGAM
ATTTGALPAQ PLPLSVPSSL AQAQTQLGPQ PEVTPKRQVL A
```

MONO MW: **243468**, pI: **5.18**
Database = **/home/sorcerer/fasta/RefSeqHuman_082508.fasta**
Links: Google Google Scholar NCBI UniProtKB

Position	Mass	Peptide
55-61	803.4	CTQLPSR
62-75	1486.67	EAACLCPPGWVGER
91-103	1233.61	GVCQSSVVAGTAR
134-141	789.34	CSVGPDGR
142-153	1326.58	FLCSCPPGYQGR
333-344	1275.55	VASFYCACPMGK
545-558	1550.6	NVDDCSPDPCHHGR
579-587	1067.4	CESQVDECR
1042-1050	913.5	EAAAQIGVR
1051-1076	2825.15	LEQLCQAGGQCVDEDSSHYCVCPEGR
1202-1210	1051.41	CEADINECR
1221-1231	1163.5	DCLQDPGGGFR
1232-1242	1146.51	CLCHAGFSGPR
1295-1309	1653.8	ELQCPVGVPCQQTPR
1313-1325	1246.53	CACPPGLSGPSCR
1361-1371	1148.49	CACAQGWTGPR
1372-1386	1554.7	CEAPAAAPEVSEEPR
1427-1435	1135.49	QCEALQCWR
1466-1473	952.43	TCNPVYEK
1474-1483	1153.46	YCADHFADGR
1838-1848	1138.61	TGETALHLAAR
2070-2078	851.46	AGLGPQGPR

Supplementary Figure 1. **Mass spectrometry identification of NOTCH family proteins as Act1-interacting proteins.** HeLa cell lysates were immunoprecipitated with anti-Act1 antibody followed by mass spectrometry analysis of co-precipitated proteins. Co-immunoprecipitated proteins were identified by mass spectrometry. Shown are the NOTCH1, NOTCH2 and NOTCH3 peptides identified in the analysis.

Supplementary Figure 2.

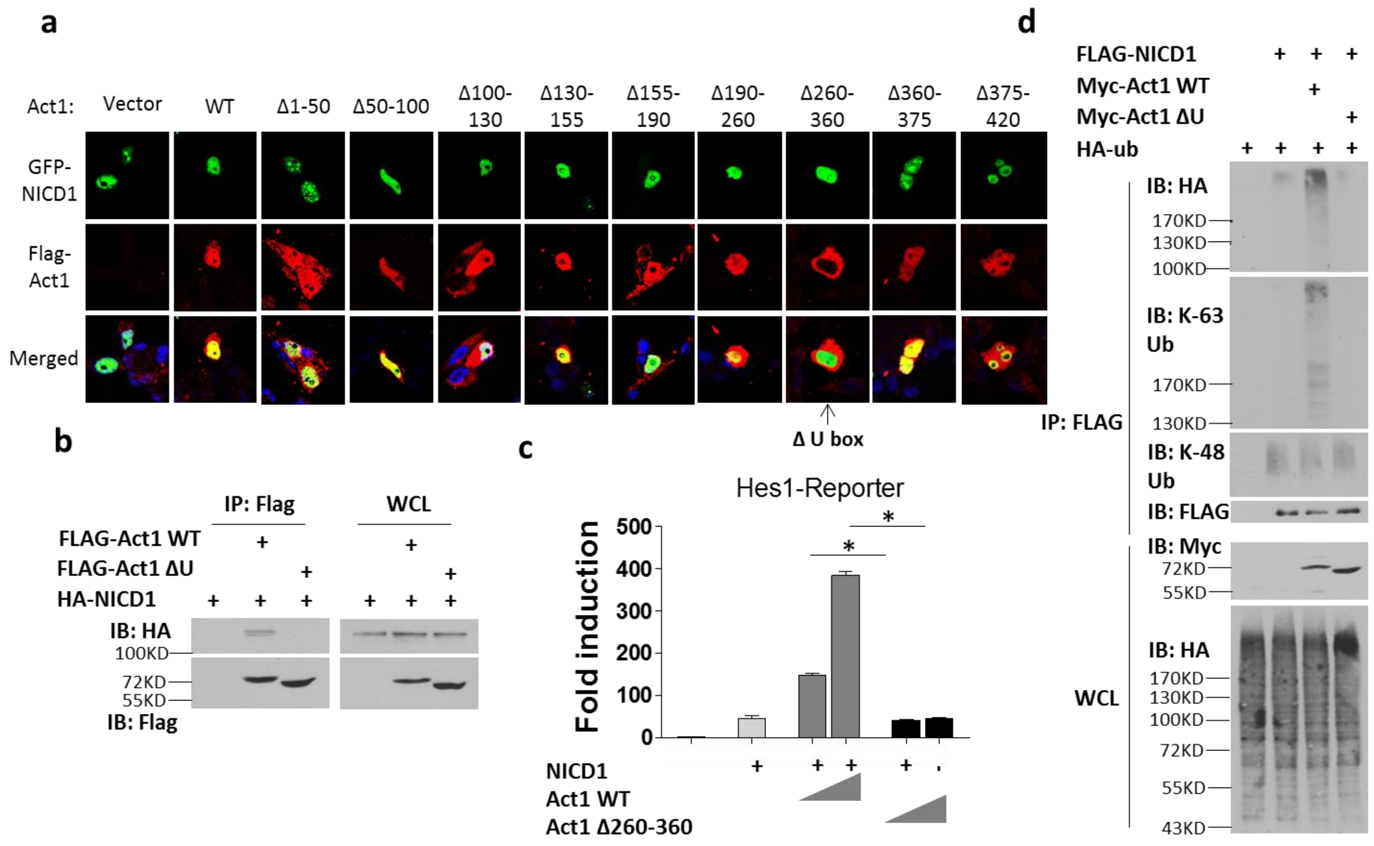

Supplementary Figure 2. E3 ligase activity of Act1 is indispensable for engaging NOTCH pathway. (a) HeLa cells were transfected with GFP-NICD and indicated Flag tagged wild-type Act1 or Act1 deletion mutants. Transfected cells were fixed and stained with anti-Flag antibody followed by analysis with confocal microscope under a 63X objective. Arrow indicates U box deletion mutant of Act1 (*Δ 260-360*) (b) HeLa cells were transfected with HA-NICD and vector, wild-type Act1 or U-box deletion mutant of Act1 (Act1 *Δ*U). Cell lysates were immunoprecipitated with anti-FLAG antibody, followed by immunoblot analysis for indicated proteins. IB: immunoblotting; IP: Immunoprecipitation, WCL: whole cell lysates. (c) HeLa cells were transfected with Hes1–luciferase reporter (100ng) alone or in combination with the different amounts of wild-type Act1 (200ng and 500ng) or Act1 *ΔU* (200ng and 500ng) and NICD1 (200ng). Data is reported as fold induction of luciferase activity in indicated transfection over Hes1-reporter transfection alone. Data are repeated two different experiments. (d) HeLa cells were transfected with HA-Ub in combination with wild-type myc-Act1, myc-Act1 *ΔU* and FLAG-NICD as indicated. Cell lysates were immunoprecipitated with anti-FLAG antibody, followed by immunoblot analysis for indicated proteins. IB: immunoblotting; IP: Immunoprecipitation, WCL: whole cell lysates. Data are representative of two independent experiments.

Supplementary Figure 3.

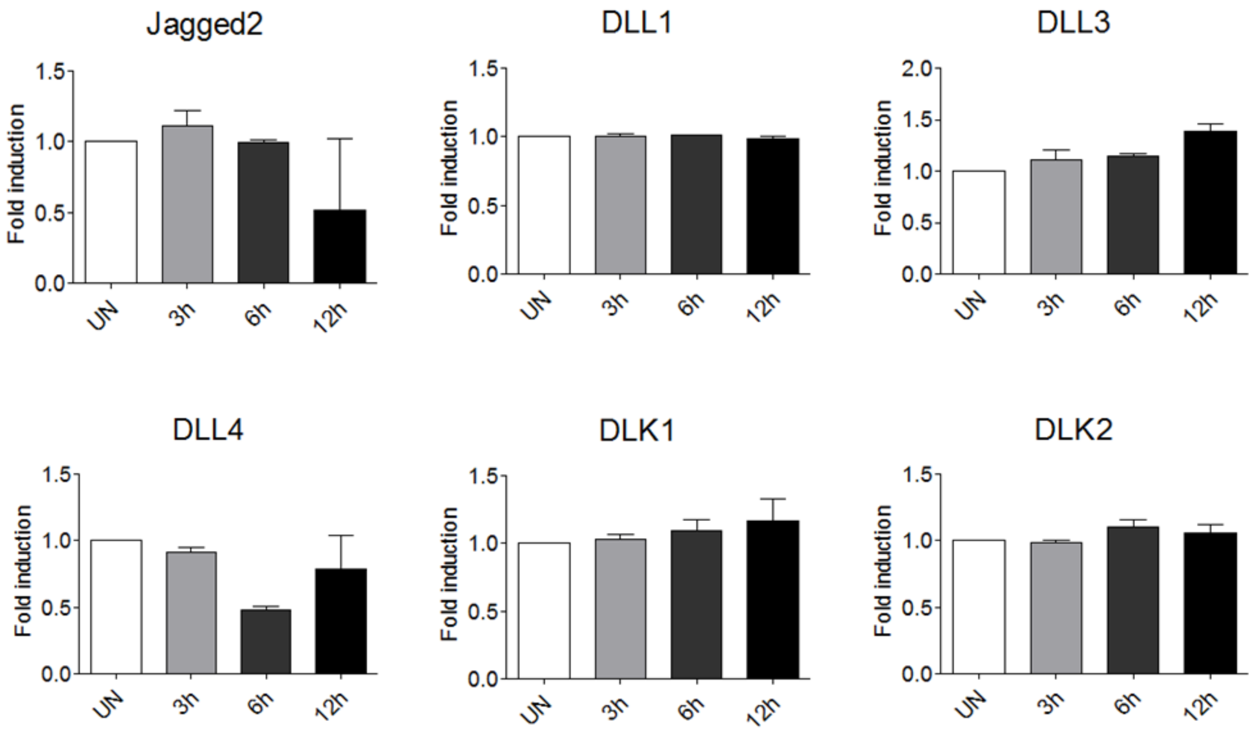

Supplementary Figure 3. IL-17 stimulation does not induce NOTCH1 ligand expression in astrocytes. Astrocytes were stimulated with IL-17 (50ng ml^{-1}) for indicated time. Stimulated cells were subjected to RT-PCR analysis for the expression of indicated NOTCH ligand. Data are representative of two experiments.

Supplementary Figure 4.

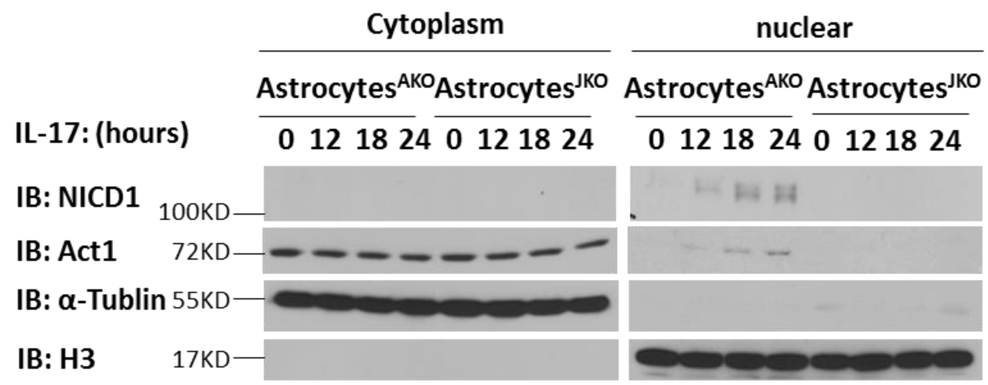

Supplementary Figure 4. Jagged1 expressed in astrocytes is required for IL-17-induced Act1 nuclear translocation and OPCs maturation. OPCs co-cultured with Act1 KO or Jagged1 KO astrocytes were left untreated or treated with IL-17 (50ng ml^{-1}) for indicated time, followed by cell fractionation for cytoplasmic and nuclear proteins. Cell fractions were analyzed by immunoblot analysis for indicated proteins. Data are representative of two independent experiments.

Supplementary Figure 5.

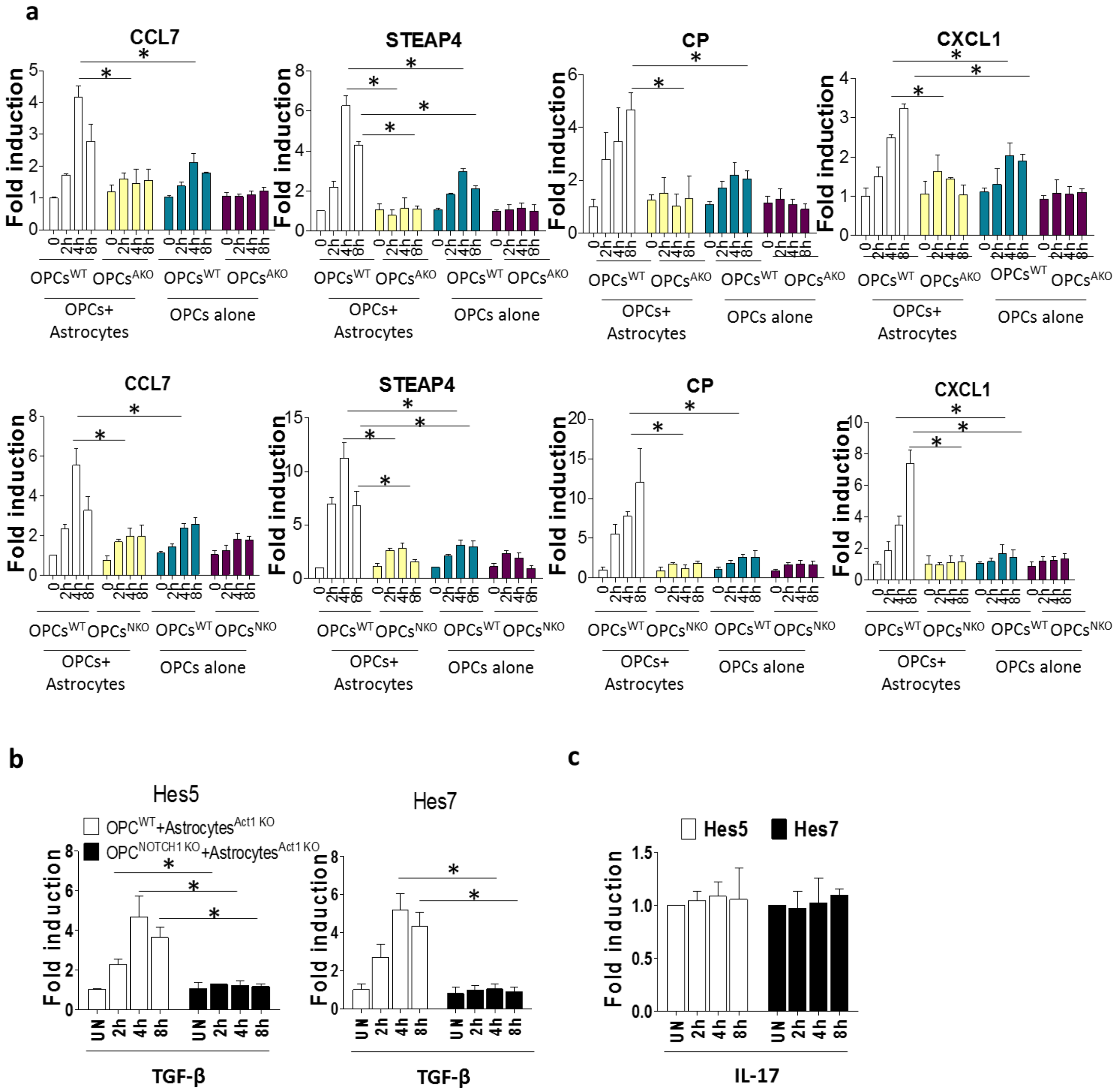

Supplementary Figure 5. Differential regulation of gene expression by canonical IL-17 pathway and IL-17-NOTCH axis. (**a**) Act1- and NOTCH1 -dependent genes. Wild-type, Act1 KO or NOTCH1 KO OPCs co-cultured with Act1 KO astrocytes were treated with IL-17 for indicated time, followed by real-time PCR analysis of different gene induction. (**b**) Wild-type or NOTCH KO OPCs co-cultured with Act1 KO astrocytes were treated with TGFβ for indicated time, followed by real-time PCR analysis of different gene induction. **(c)** OPCs co-cultured with Act1 KO astrocytes were stimulated with IL-17 for indicated time, followed by RT-PCR analysis for indicated genes. Error bars represent SEM of technical replicates. *P < 0.05 based on Mann-Whitney U test. Data are representative of three independent experiments.

Supplementary Figure 6

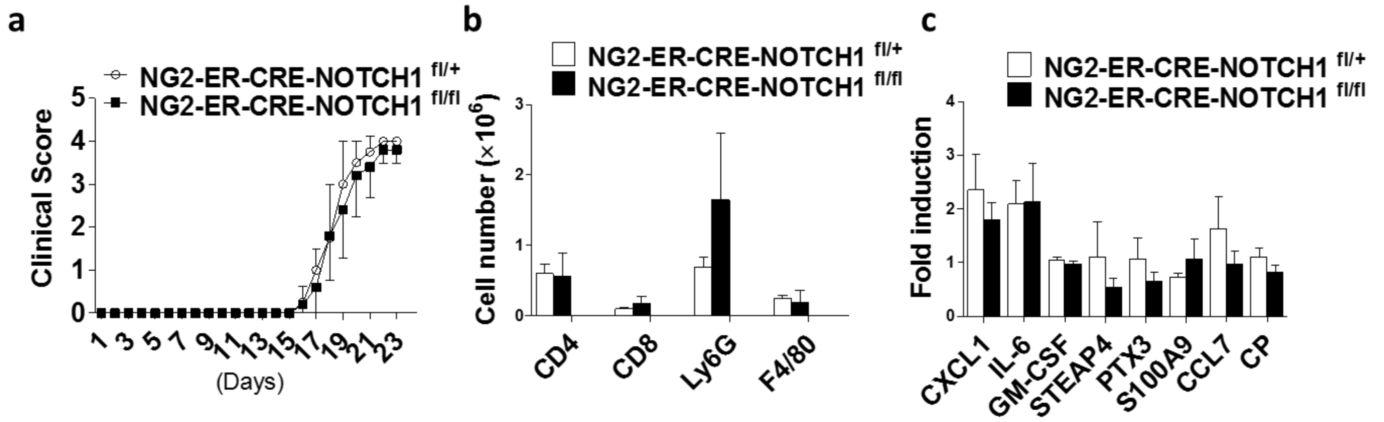

Supplementary Figure 6. NOTCH1 deficiency in NG2+ cells does not affect Th1-induced EAE.

(a) Mice (n=15) of indicated genotypes were adoptively transferred with MOG-reactive Th1 cells. Clinical scores of EAE symptoms are graphed over experimental time course. **(b)** Infiltrating cells from the brain of mice with Th1 induced EAE (n=5) were analyzed by flow cytometry at the peak of the disease. **(c)** RT-PCR analysis of inflammatory gene expression in spinal cords of mice (n=5) with Th1 induced EAE. All error bars represent SEM of biological replicates. *: $p<0.05$ based on Mann-Whitney U test. Data are representative of three independent experiments.

Supplementary Figure 7

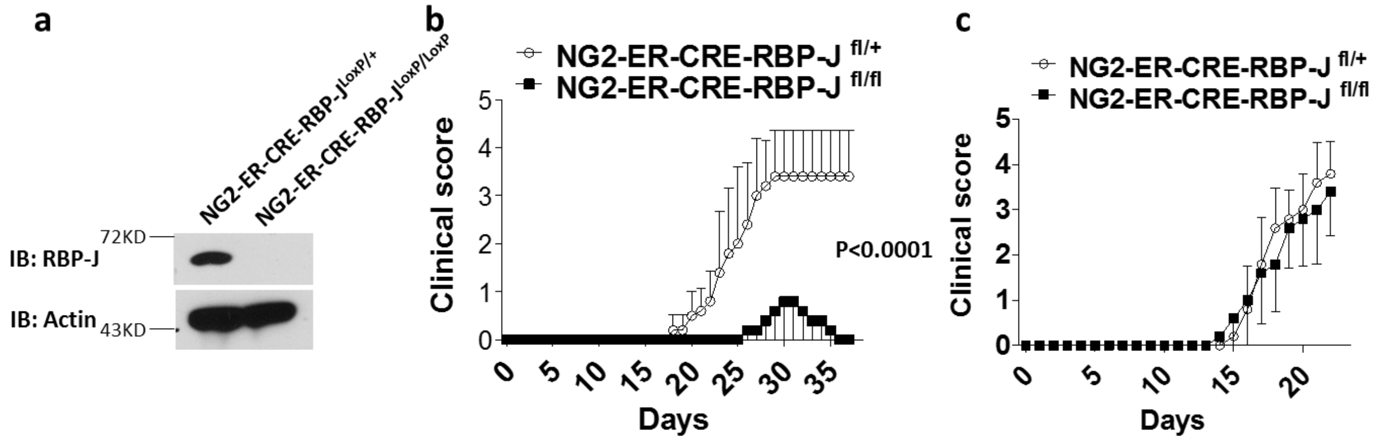

Supplementary Figure 7. Ablation of RBP-J in OPCs ameliorates Th17- but not Th1-induced EAE. (a) Immunoblot analysis of RBP-J expression in NG2$^+$ cells sorted from brains of indicated mice. **(b)** Mice of indicated genotypes were adoptively transferred with MOG-reactive Th17 cells (n = 5) to induce EAE. Clinical score of symptoms are graphed over the experimental time course. **(c)** Mice of indicated genotypes were adoptively transferred with MOG-reactive Th1 cells (n = 5) to induce EAE. Clinical score of symptoms are graphed over the experimental time course. Data are representative of three independent experiments.

Supplementary Figure 8

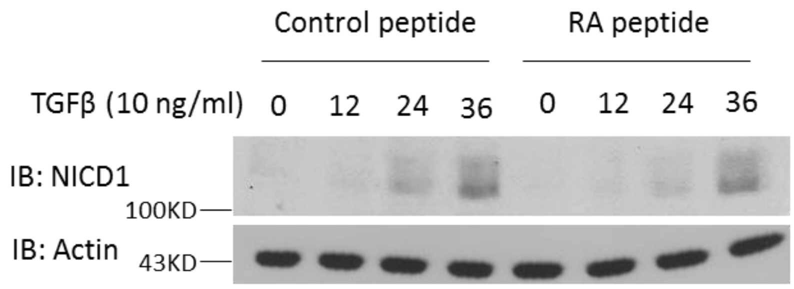

Supplementary Figure 8. IL-17RA decoy peptide does not block TGFβ-induced NOTCH1 activation in OPC-astrocyte co-culture. OPCs co-cultured with Act1 KO astrocytes were left untreated or treated with recombinant TGFβ (10ng ml^{-1}) for indicated time, followed by immune blot for NICD1 and Actin. Data are representative of two independent experiments.

Supplementary Figure 9. Uncropped image of films for all the Figures.

Figure 1a

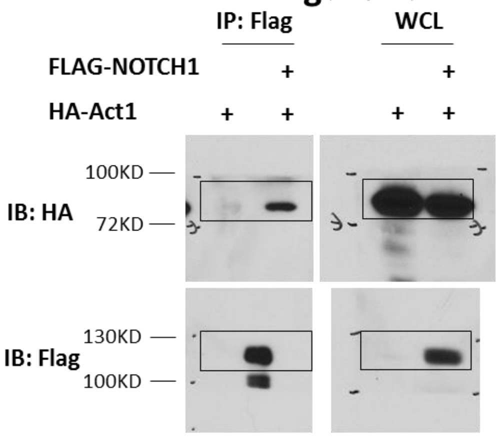

Figure 1f (upper)

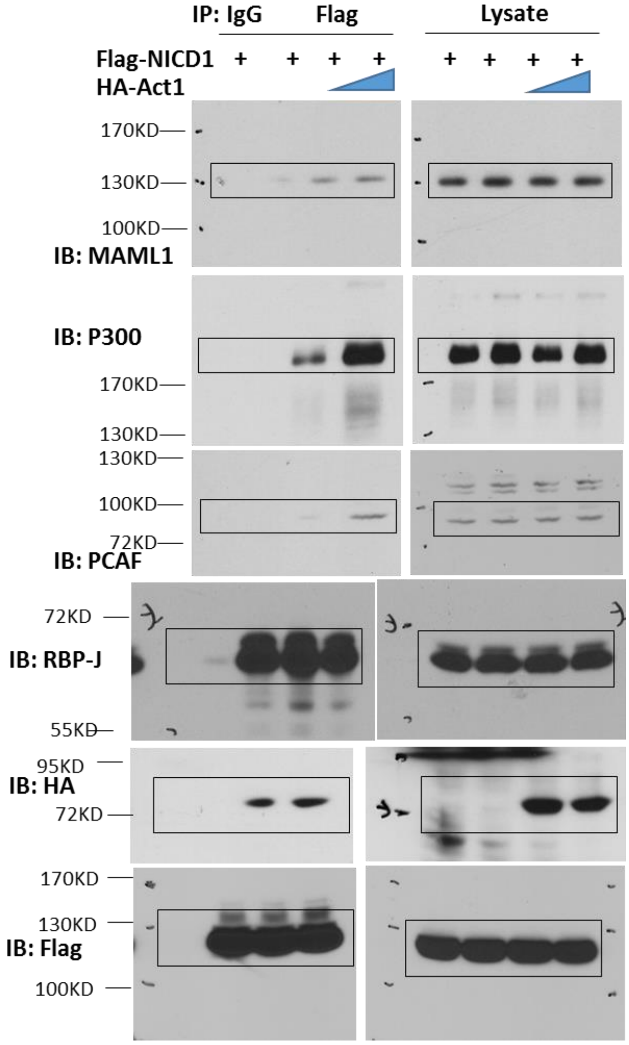

Figure 1b

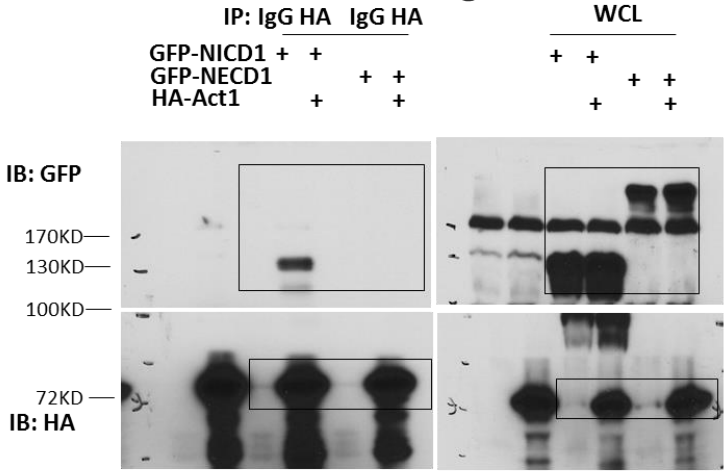

Figure 1f (Lower)

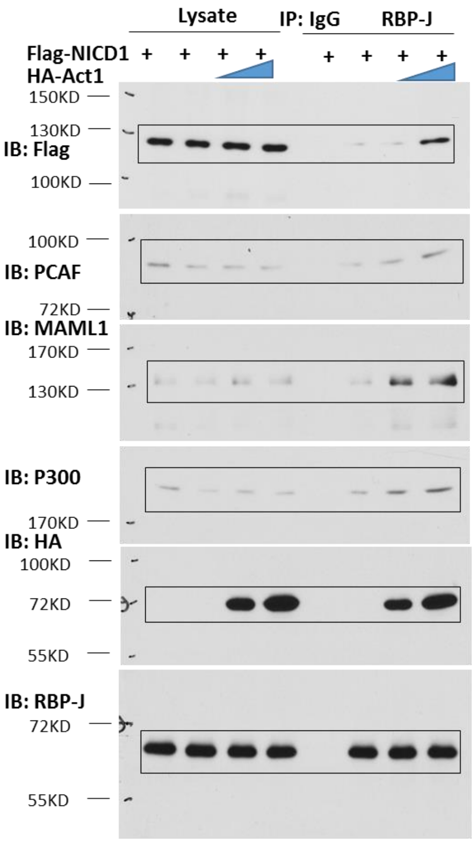

Figure 2a

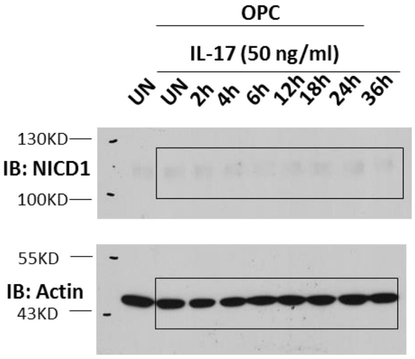

Figure 2c

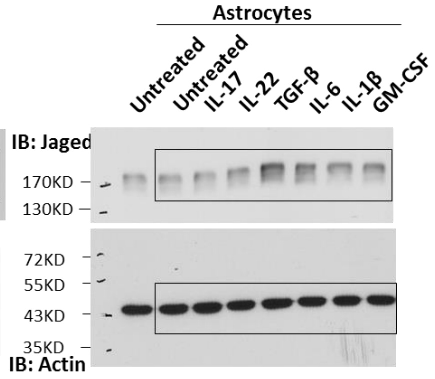

Figure 2b

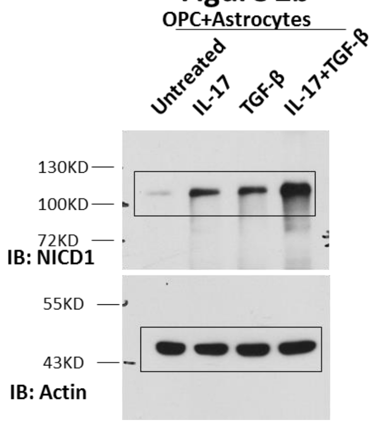

Figure 2d (left)

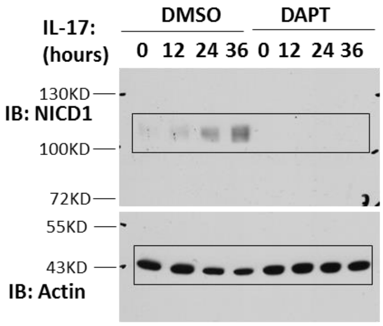

Figure 2d (left)

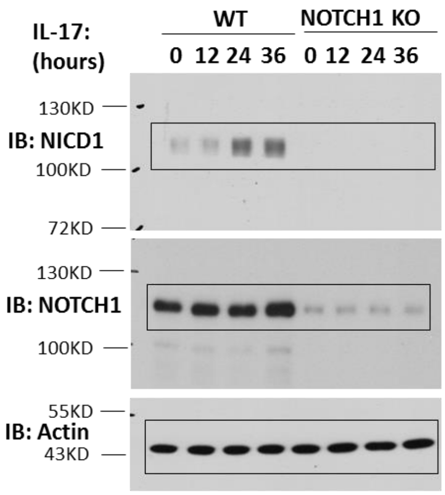

Figure 2f

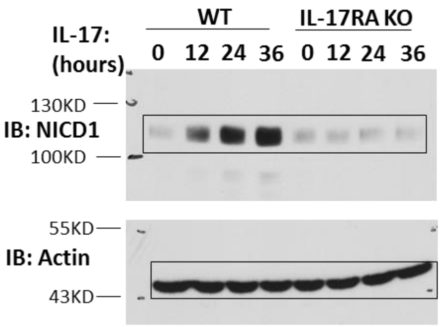

Figure 2h

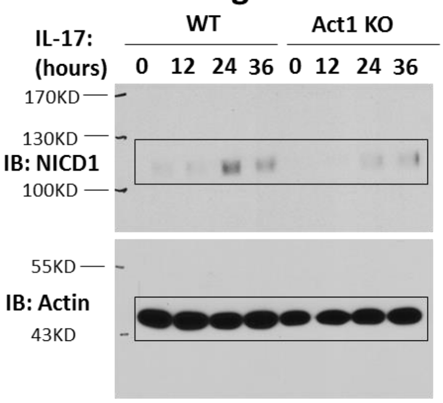

Figure 2e

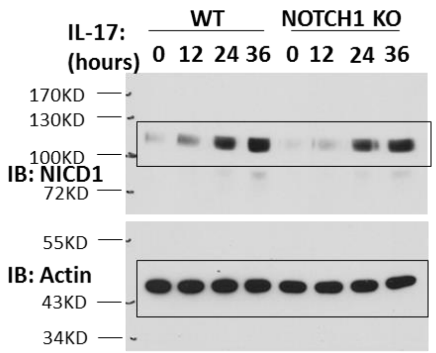

Figure 2g

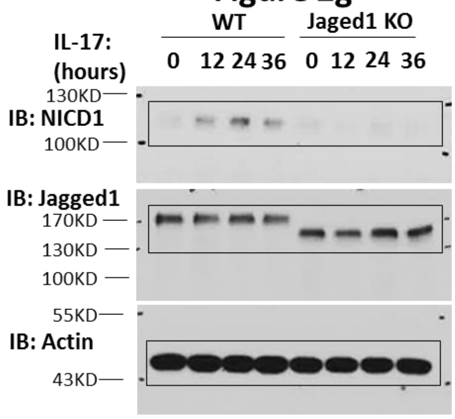

Figure 2i

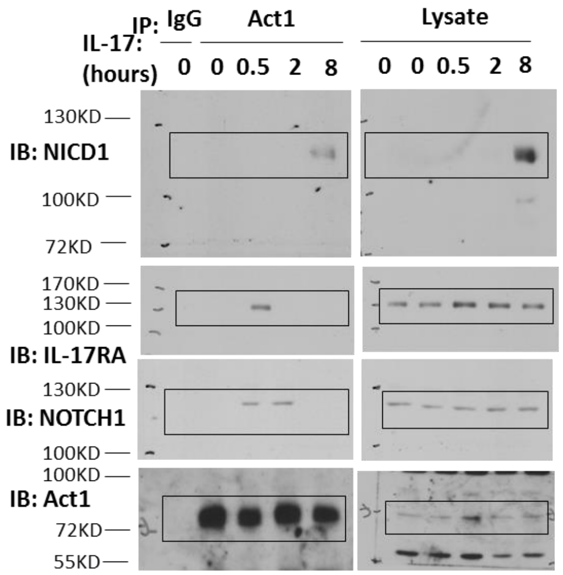

Figure 3a

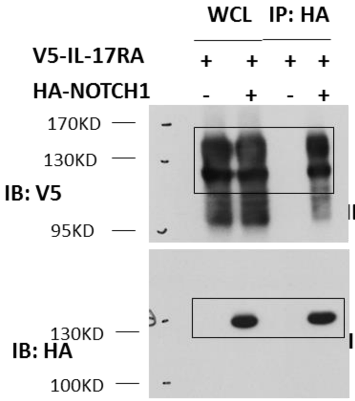

Figure 3c left

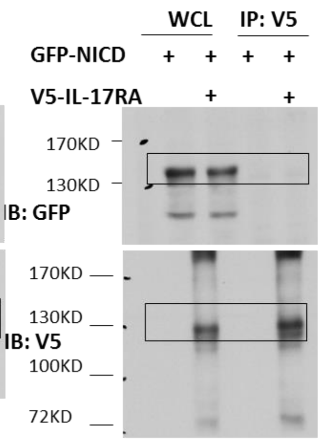

Figure 3d

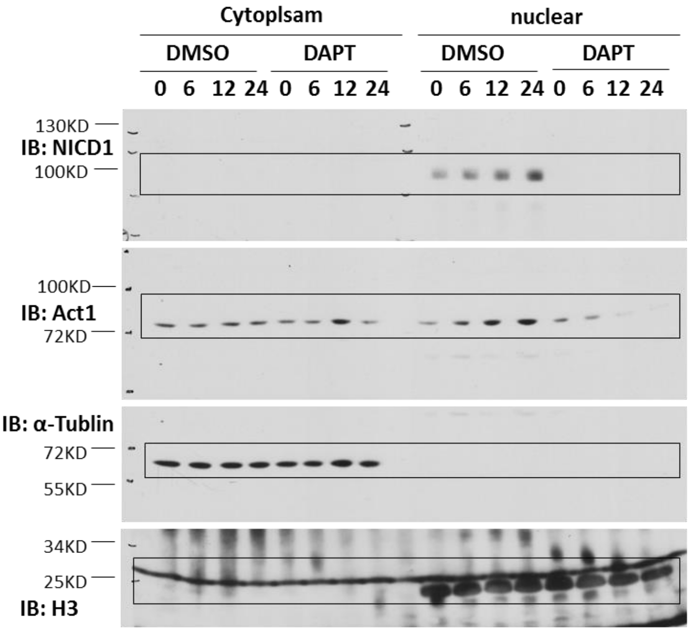

Figure 3c right

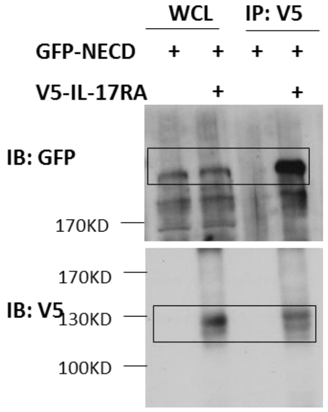

Figure 3c

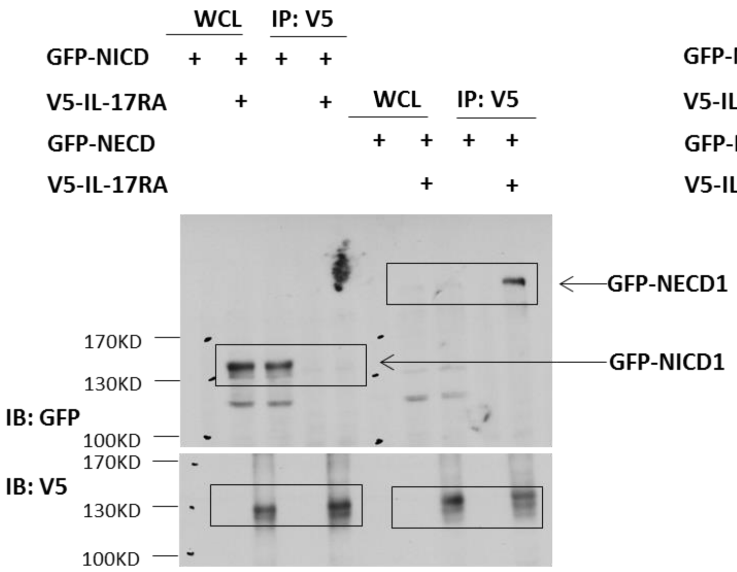

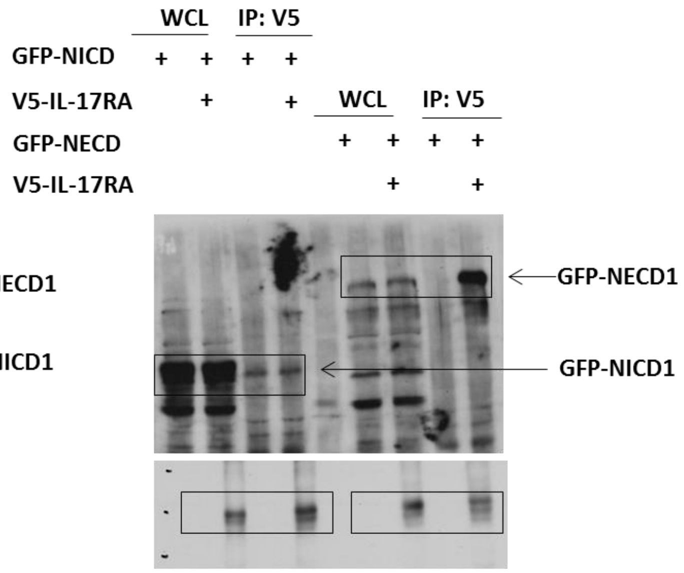

Short exposure **Longer exposure**

Figure 3e

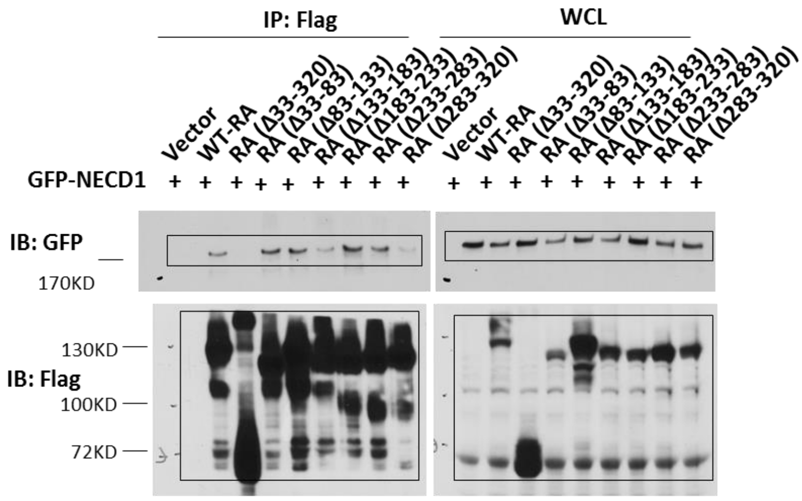

Figure 6a

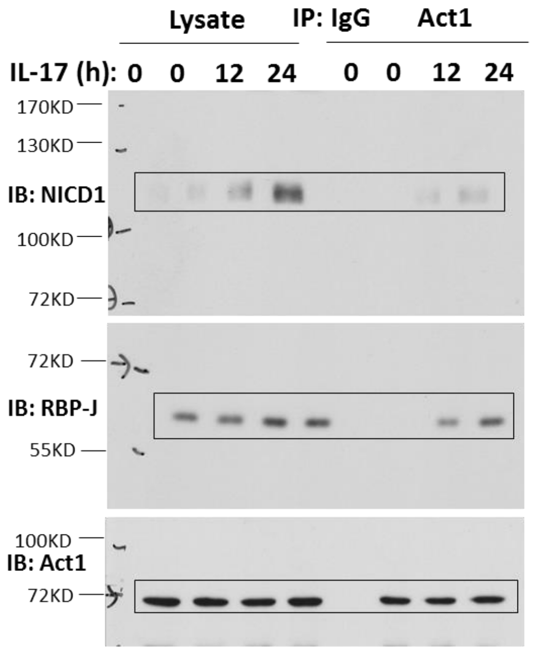

Figure 6d

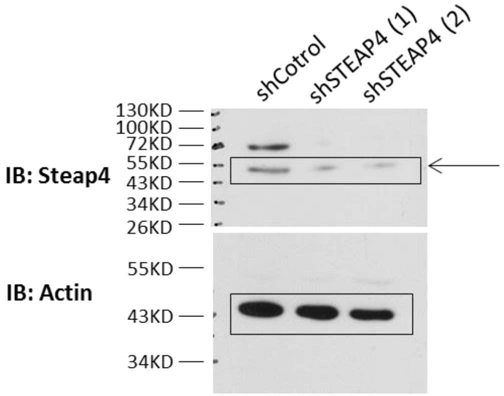

Figure 9a

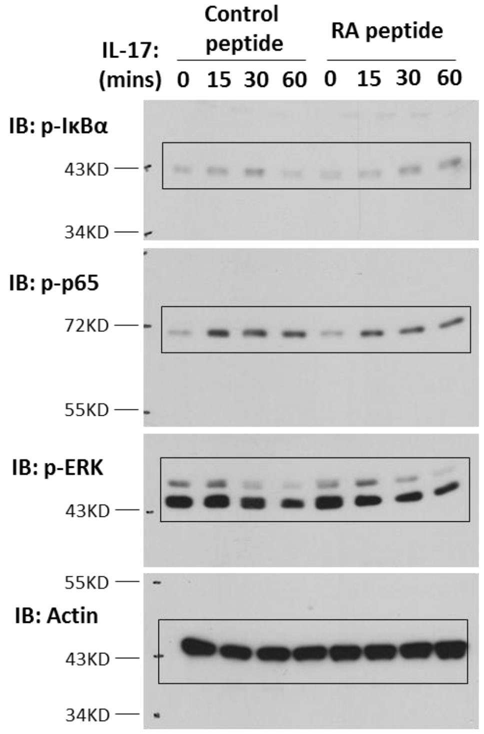

Figure 9b

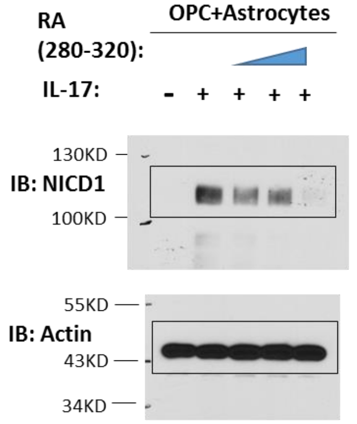

Figure 9c

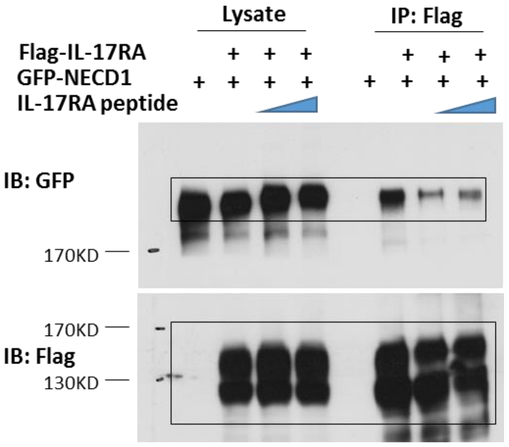

Sup Figure 2a

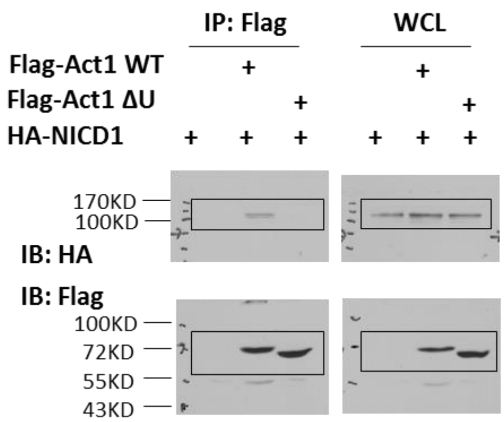

Sup Figure 2c

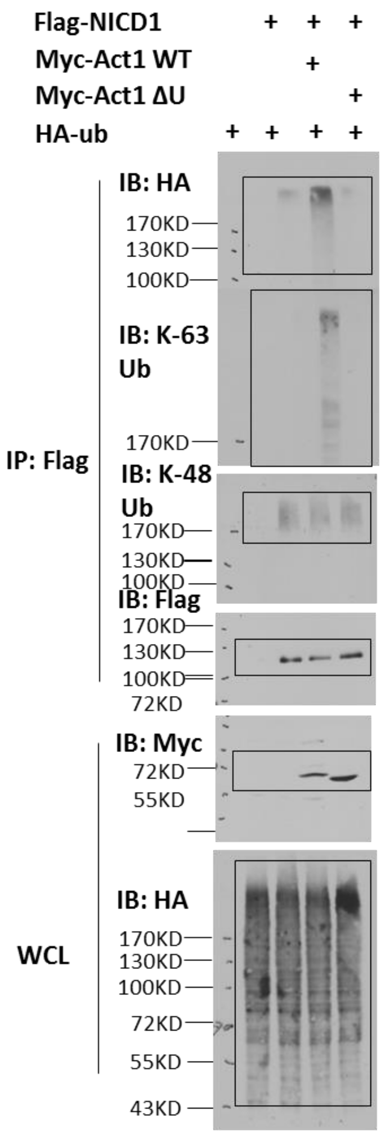

Sup Figure 7a

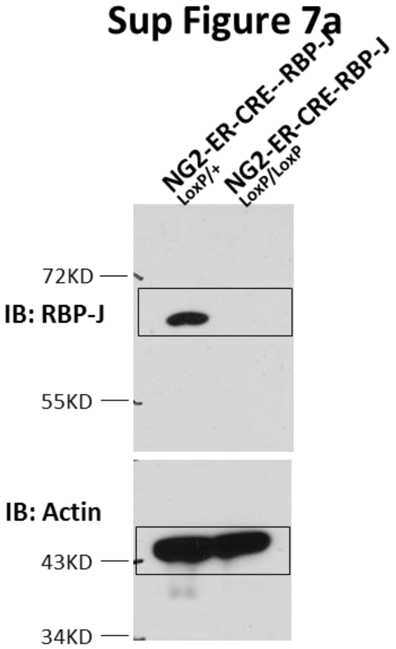

Sup Figure 4

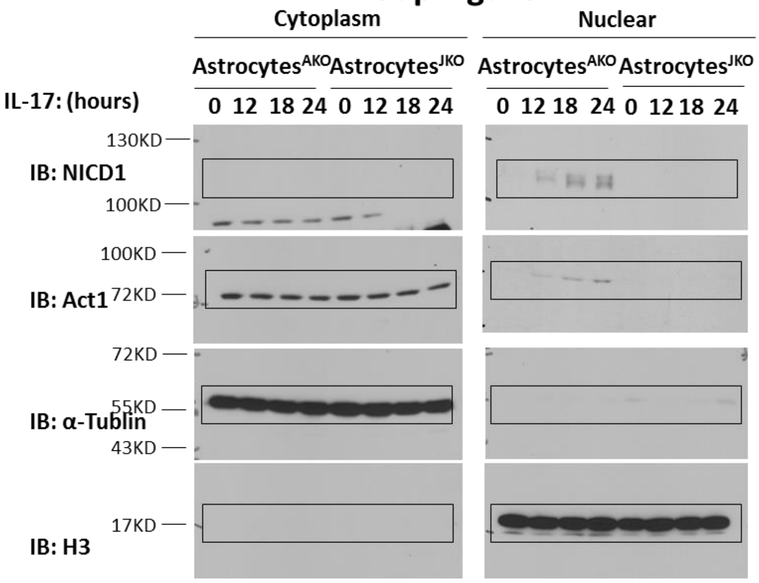

Sup Figure 8

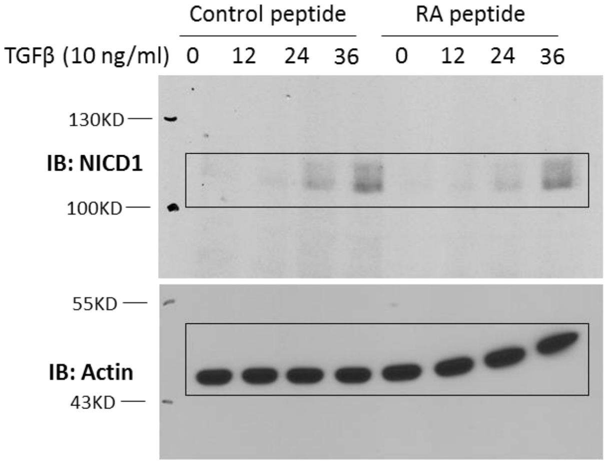

Supplementary table 1: Primer sequence

Gene name	Forward primer	Reverse Primer
Ccl7	5’-accagtagtcggtgtccctg-3’	5’-ctatagcctcctcgacccacttc-3’
Steap4	5’-gaacactagatgcaagccgg-3’	5’-gagagatccttggtccagtgg-3’
Ptx3	5’-gggtggactcctacagattgg-3’	5’-ctgagaacccgatcccagat-3’
Csf2	5’-ctaacatgtgtgcagacccg-3’	5’-gtctggtagtagctggctgtc-3’
Ccl20	5’-cacaagacagatggccgatg-3’	5’-cccttttcacccagttctgc-3’
Cxcl1	5’-ccagagcttgaaggtgttgc-3’	5’-tgaaccaagggagcttcagg-3’
Cp	5’-gtgactgtaacaagccctcg-3’	5’-agtgaagatgtccgtcccag-3’
Mmp9	5’-cgacatagacggcatccagt-3’	5’-gataggccgtgggaggtatag-3’
S100a9	5’-ggccaacaaagcaccttctc-3’	5’-tccttcttgctcagggtgtc-3’
Hes5	5’-tgcaccaggactacagcgag-3’	5’-cgctggaagtggtaaagcag-3’
Hes7	5’-catcaaccgcagcctagaag-3’	5’-cgaactccagtatctccgct-3’
Mbp	5’-cttcagaccatccaagaagacc-3’	5’-gtacttgctgtggccaggtact-3’
Mag	5’-tcaaggagaagcagatcctagc-3’	5’-tctcagccacacaccagtattc-3’
Mog	5’-ccttctcaagagtggttcacct-3’	5’-gggtaacctttccctcactgat-3’

Gene name	Forward primer	Reverse Primer
IL-17RA Δ33-320	5’-CTGGGCGTGCTGGCCCCGGGTGGCGCCTCCGTGTACTGGTTCATCACGGGCATCTCCATCC-3’	5’-GGATGGAGATGCCCGTGATGAACCAGTACACGGAGGCGCCACCCGGGGCCAGCACGCCCAG-3’
IL-17RA Δ33-83	5’-CGTGCTGGCCCCGGGTGGCGCCTCCCACACCCAACAAGGAGACCTGTTCC-3’	5’-GGAACAGGTCTCCTTGTTGGGTGTGGGAGGCGCCACCCGGGGCCAGCACG-3’
IL-17RA Δ83-133	5’-GGACCTGCAGATCCAGCTGCACTTTAAACTGAGGCATCACCACAGGCGGT-3’	5’-ACCGCCTGTGGTGATGCCTCAGTTTAAAGTGCAGCTGGATCTGCAGGTCC-3’
IL-17RA Δ133-183	5’-GTGCGTCAGGTTTGAGTTTCTGTCCGACTGTGAGCACGCCAGGATGAAGG-3’	5’-CCTTCATCCTGGCGTGCTCACAGTCGGACAGAAACTCAAACCTGACGCAC-3’
IL-17RA Δ183-233	5’-CCAGTCCAAGAATTTCCTTGTGCCTCTGACCAGTTTTCCGCACATGGAGA-3’	5’-TCTCCATGTGCGGAAAACTGGTCAGAGGCACAAGGAAATTCTTGGACTGG-3’
IL-17RA Δ233-283	5’-CGAATCTACCCATTACCAGATCCTGCAGCCCTTCTTCAGCAGCTGCCTCA-3’	5’-TGAGGCAGCTGCTGAAGAAGGGCTGCAGGATCTGGTAATGGGTAGATTCG-3’
IL-17RA Δ283-320	5’-GGTGCTGTCGCCACCAAGTGCAGATCGTGTACTGGTTCATCACGGGCATCT-3’	5’-AGATGCCCGTGATGAACCAGTACACGATCTGCACTTGGTGGCGACAGCACC-3’

Gene name	Forward primer	Reverse Primer
Act1 Δ50-100	5'-AGAACCACCTGCTCCAAATATAAGGGAAGACAGTTTCTGCAGGAGACACC-3'	5'-GGTGTCTCCTGCAGAAACTGTCTTCCCTTATATTTGGAGCAGGTGGTTCT-3'
Act1 Δ100-130	5'-GCACTCAAGTTCTGGAGGACGTTGGAGCCCTCCCTGCAGA-3'	5'-TCTGCAGGGAGGGCTCCAACGTCCTCCAGAACTTGAGTGC-3'
Act1 Δ130-155	5'-GCGAGCCTGCGTCTGAGTCTGCGGCTTCTCCTGACACTGG-3'	5'-CCAGTGTCAGGAGAAGCCGCAGACTCAGACGCAGGCTCGC-3'
Act1 Δ155-190	5'-GTAATCAATGGCTGGTATCTCAGCTTCAGCCTCACAGGAACCGAGCAGGCC-3'	5'-GGCCTGCTCGGTTCCTGTGAGGCTGAAGCTGAGATACCAGCCATTGATTAC-3'
Act1 Δ190-260	5'-CGGTAGCCAGGAGATGGTGCAACGGCCTCCCAATCTTTCCCCACATGCTC-3'	5'-GAGCATGTGGGGAAAGATTGGGAGGCCGTTGCACCATCTCCTGGCTACCG-3'
Act1 Δ260-360	5'-GAGGTATCCAGCATGTGCACAGATGGCTGGTGCTCCTGGGGAGTCCTTGG-3'	5'-GCTGGGGAACCTGTGGATTAGGTGGCTGGTGA-3'
Act1 Δ360-375	5'-TCACCAGCCACCTAATCCACAGGTTCCCCAGC-3'	5'-AGATGCCCGTGATGAACCAGTACACGATCTGCACTTGGTGGCGACAGCACC-3'
Act1 Δ375-420	5'-GGAGTCCTTGGAGTGCCCTGCAGAGCTGGCTATGGAGGTGGTGAAATTCGTGA-3'	5'-TCACGAATTTCACCACCTCCATAGCCAGCTCTGCAGGGCACTCCAAGGACTCC-3'

Supplementary table 2 : ChIP primer sequence

Gene name	Forward primer	Reverse Primer
CXCL1 ChIP	5’ -TTGGCTCCTGAGTCTAAGAA-3’	5’ -GTAAAACTGAGTCTTTGAATATT-3’
S100A9 ChIP	5’ -GGTTGCCAACTGTGCTTCCA-3’	5’ -ATGGCCAACAAAGCACCTTCT-3’
PTX3 ChIP	5’ -AGGGTAGTAGCCCTGCCATC-3’	5’ -GAACCACTCCCAGAGGATACC-3’
STEAP4 ChIP	5’ -TGGCATCCATTTAGGTAACAT-3’	5’ -CTCTGTGTTCTCCAGGGCT-3’
CCL7 ChIP	5’ -CCCTGCCCTTGCCTAATACTC-3’	5’ -AAGTGTCCAGGCACATAGGC-3’
CP ChIP	5’ -TATACATTTAGATACATGCCAC-3’	5’ -GAATGATTTTACTCAACTGGAA-3’
Control ChIP	5’ -TCGATATCCACGTGACATCCA-3’	5’ -GCAGCATTTTTTTACCCCCTC-3’
